# Supplementary material for: The development of brain pericytes requires expression of the transcription factor nkx3.1 in intermediate precursors
Source: PLoS Biol. 2024 Apr 29;22(4):e3002590. doi: 10.1371/journal.pbio.3002590 (PMC11081496; doi:10.1371/journal.pbio.3002590)
Supplement: S1 Fig — (A, D, G, J). Schematics of lineage tracing strategy showing how Tg(tbx6:Cre) or Tg(sox10:Cre) drivers crossed to the Tg(loxp-stop-loxp-H2B-GFP) reporter labels progeny with nuclear GFP. Pericytes TgBAC(pdgfrb:Gal4FF) or nkx3.1-expressing cells TgBAC(nkx3.1:Gal4) +Tg(UAS:ntr:mCherry) label cytoplasm red. (B-L) All images are dorsal views of embryonic mid or hind brains at 75 hpf, as marked. (B, C, E, F) Mesodermal lineage trace of pericytes (B, C) and nkx3.1 cells (E, F). (H, I, K, L) Neural crest lineage trace of pericytes (H, I) and nkx3.1 cells (K, L). Arrowheads indicate double positive pdgfrβ or nkx3.1 cells. Scale bar is 50 μm. (PDF) [file pbio.3002590.s007.pdf]

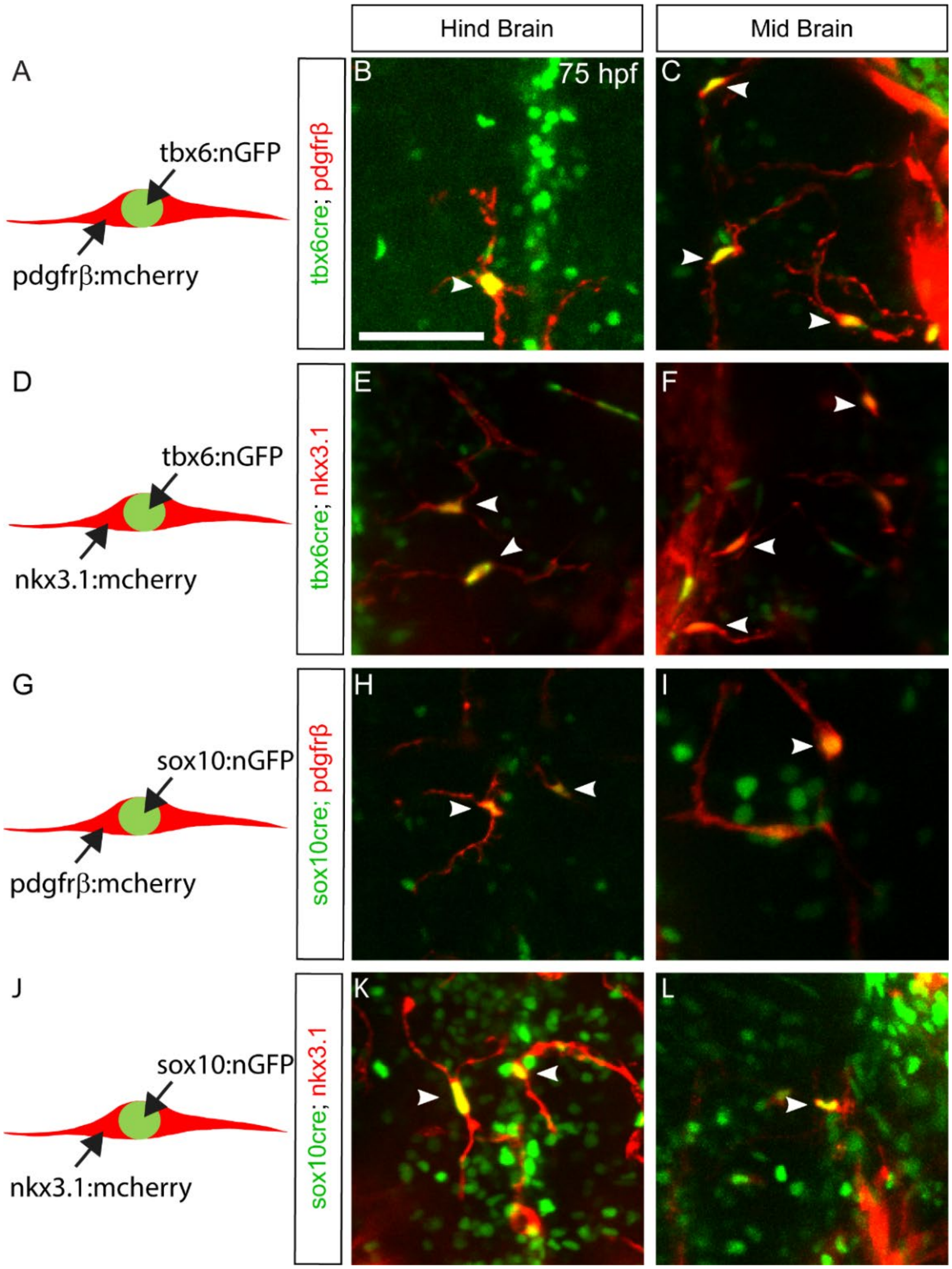

S1 Fig: Lineage tracing of nkx3.1-expressing cells and pericytes at 75 hpf. (A, D, G, J) Schematics of lineage tracing strategy showing how Tg(*tbx6:Cre)* or Tg(*sox10:Cre*) drivers crossed to the *Tg(loxp-stop-loxp-H2B-GFP*) reporter labels progeny with nuclear GFP. Pericytes *TgBAC(pdgfrb:Gal4FF) or nkx3.1-expressing cells TgBAC(nkx3.1:Gal4)* + *Tg(UAS:ntr:mCherry)* label cytoplasm red. (B-L) All images are dorsal views of embryonic mid or hind brains at 75 hpf, as marked. (B, C, E, F) Mesodermal lineage trace of pericytes (B, C) and Nkx3.1 cells (E, F). (H, I, K, L) Neural crest lineage trace of pericytes (H,I) and Nkx3.1 cells (K, L). Arrowheads indicate double positive *pdgfrβ* or *nkx3.1* cells. Scale bar is 50μm.
