# Supplementary material for: The development of brain pericytes requires expression of the transcription factor nkx3.1 in intermediate precursors
Source: PLoS Biol. 2024 Apr 29;22(4):e3002590. doi: 10.1371/journal.pbio.3002590 (PMC11081496; doi:10.1371/journal.pbio.3002590)
Supplement: S2 Fig — (PDF) [file pbio.3002590.s008.pdf]

A. DNA sequence around mutation

Nkx3.1^{wildtype}: CGGGGGGAGGCGGGAAAAAGAAGCGGTCGCGCGCCGCGT

Nkx3.1^{ca116}: CGGGGGGAGGCGGTCGCGCGCCGCGT

B. Protein sequence (teal= homeobox, grey = out of frame translation)

```
Nkx3.1wt       MATSNKQLTSFFIEDILSLKEDKKDEDSCNAESDRDDSTDRQTDSADTCRTSEGKTVSST
Nkx3.1ca116    MATSNKQLTSFFIEDILSLKEDKKDEDSCNAESDRDDSTDRQTDSADTCRTSEGKTVSST

Nkx3.1wt       EMTGGGGKKKRSRAAFTHLQVLELEKKFSRQRYLSAPERTHLASALHLTETQVKIWFQNR
Nkx3.1ca116    EMTGGGGRAPRSRTCRFWSWRRSSAVSGT*

Nkx3.1wt       RYKTKRRQLTTEHSKDYFQKSNAAAMAATEEDFFRASLLATVYKSSPYRPYVVDLHGLSM

Nkx3.1wt       WRPAL*
```

C. Schematic of protein domains

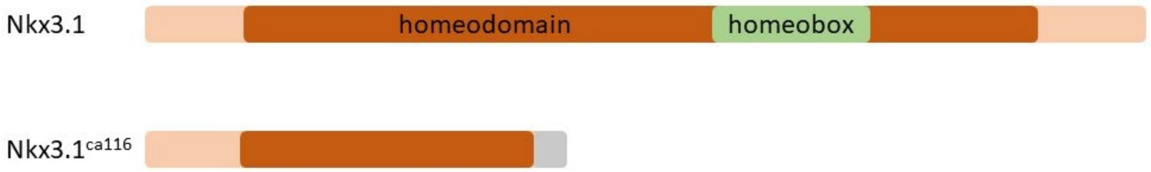

S2 Fig: Nkx3.1^{ca116} mutation characterization
