# Supplementary material for: The development of brain pericytes requires expression of the transcription factor nkx3.1 in intermediate precursors
Source: PLoS Biol. 2024 Apr 29;22(4):e3002590. doi: 10.1371/journal.pbio.3002590 (PMC11081496; doi:10.1371/journal.pbio.3002590)
Supplement: S3 Fig — (A, B) Dorsal views of embryonic brain of zygotic nkx3.1 mutants showing no change in brain pericyte numbers as compared to wild type. Pericytes (green, arrowheads) are labelled with TgBAC(pdgfrβ:GFP) and vessels (red) are labelled with Tg(kdrl:mCherry). (C) Quantitation of pericyte numbers shows no significance using one-way ANOVA with Tukey’s test. (n = 8 wild types, 17 heterozygotes, and 10 mutants). Scale bar is 50 μm. The data underlying this figure can be found in S3 Table. (PDF) [file pbio.3002590.s009.pdf]

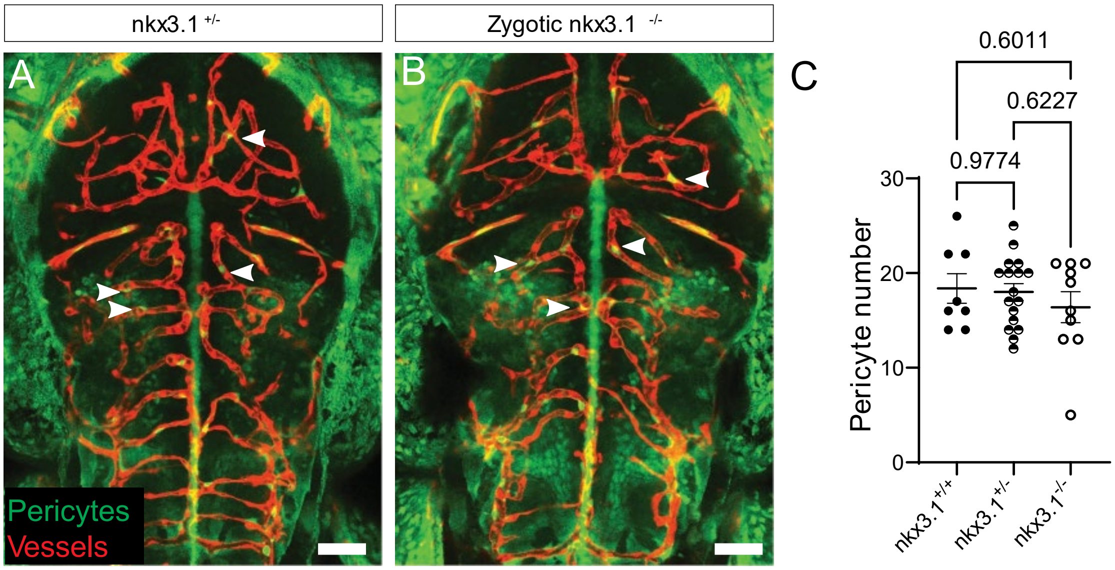

S3 Fig: Zygotic *nkx3.1* mutants have wildtype pericyte numbers at 75 hpf.

(A-B) Dorsal views of embryonic brain of zygotic *nkx3.1* mutants showing no change in brain pericyte numbers as compared to wildtype. Pericytes (green, arrowheads) are labelled with *TgBAC(pdgfrβ:GFP)* and vessels (red) are labelled with *Tg(kdrl:mCherry)*. (C) Quantitation of pericyte numbers shows no significance using one-way ANOVA with Tukey's test. (n=8 wildtypes, 17 heterozygotes and 10 mutants). Scale bar is 50 μm. The data underlying this figure can be found in Supp Table 3.
