# Supplementary material for: The development of brain pericytes requires expression of the transcription factor nkx3.1 in intermediate precursors
Source: PLoS Biol. 2024 Apr 29;22(4):e3002590. doi: 10.1371/journal.pbio.3002590 (PMC11081496; doi:10.1371/journal.pbio.3002590)
Supplement: S4 Fig — Vessel network length of the hindbrain CtAs was measured using VesselMetrics. Genotypes and treatments are labelled. No treatment or mutant significantly alters the endothelial vessel length. Statistics used a Student t test. The data underlying this figure can be found in S3 Table. (PDF) [file pbio.3002590.s010.pdf]

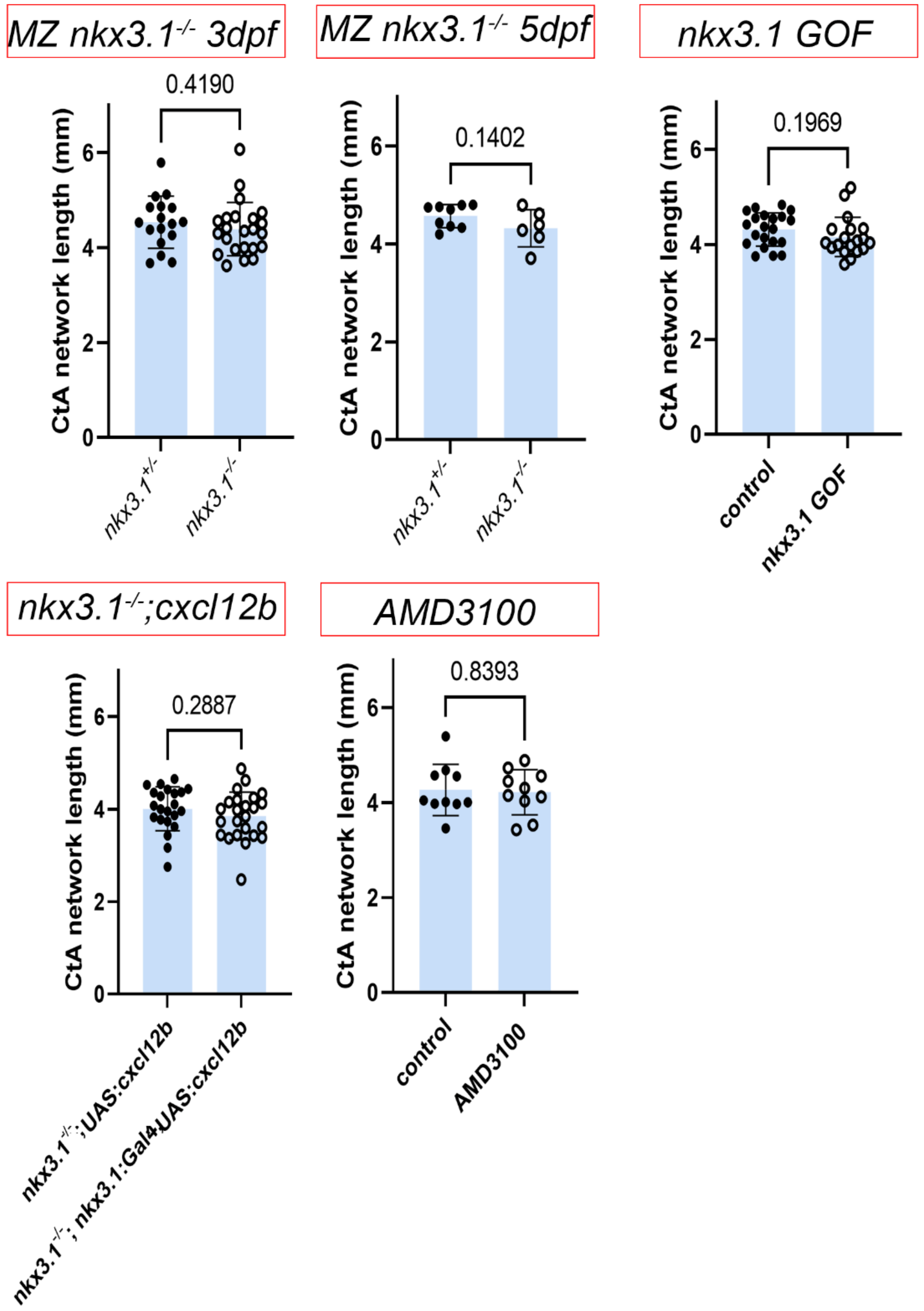

S4 Fig: Central Artery vessel network length is unchanged across multiple experimental manipulations

Vessel network length of the hindbrain CtAs was measured using VesselMetrics. Genotypes and treatments are labelled. No treatment or mutant significantly alters the endothelial vessel length. Statistics used a Student's t-test. The data underlying this figure can be found in Supp Table 3.
