# Supplementary material for: The development of brain pericytes requires expression of the transcription factor nkx3.1 in intermediate precursors
Source: PLoS Biol. 2024 Apr 29;22(4):e3002590. doi: 10.1371/journal.pbio.3002590 (PMC11081496; doi:10.1371/journal.pbio.3002590)
Supplement: S5 Fig — (A, B) Dorsal views of embryonic brain of MZ nkx3.1 mutants at 5 dpf. Pericytes (green, arrowheads) are labelled with TgBAC(pdgfrβ:GFP) and vessels (red) are labelled with Tg(kdrl:mCherry). There are significantly fewer brain pericytes (C) and reduced pericyte density (D) in nkx3.1−/− mutant as compared to nkx3.1+/− controls. Statistics used a Student t test. (n = 9 wild types, 6 mutants). Scale bar is 50 μm. The data underlying this figure can be found in S3 Table. (PDF) [file pbio.3002590.s011.pdf]

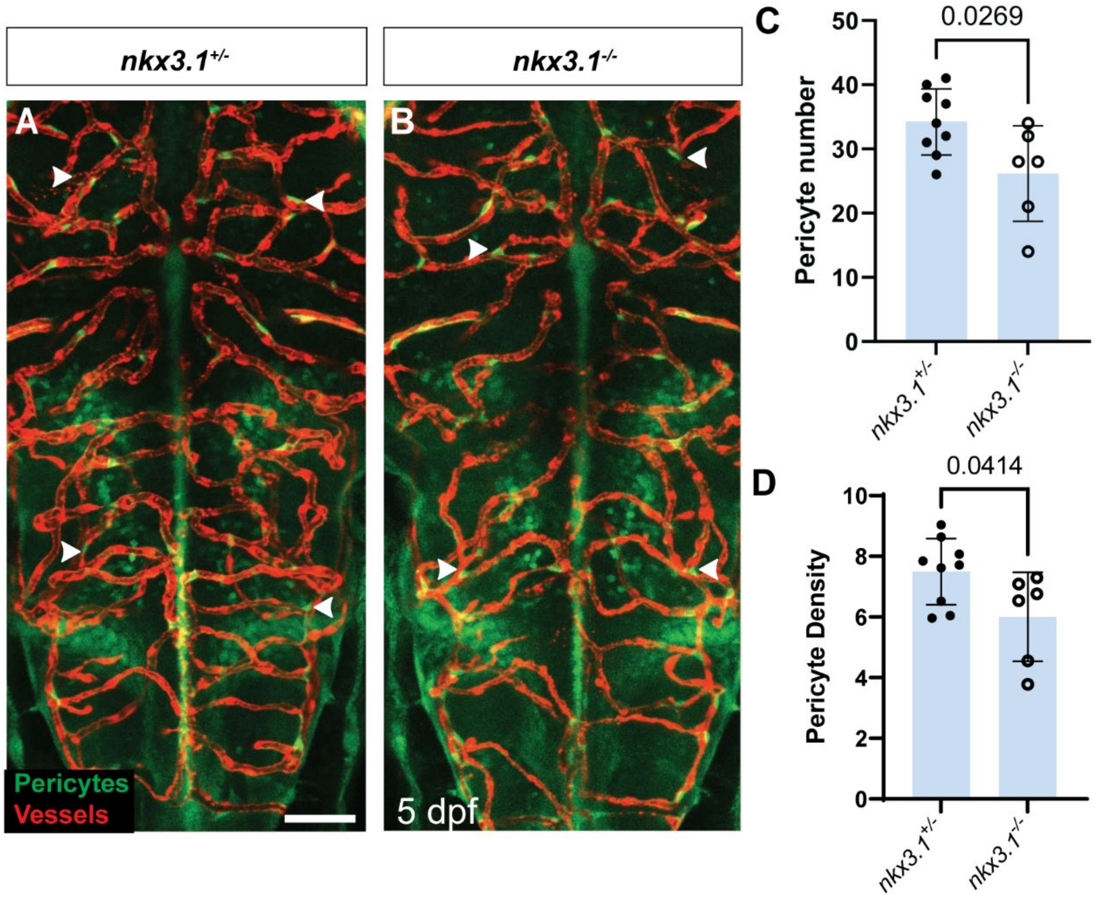

S5 Fig: Pericyte number and density is decreased at 5dpf in *nkx3.1* maternal-zygotic mutants

(A-B) Dorsal views of embryonic brain of MZ *nkx3.1* mutants at 5 dpf. Pericytes (green, arrowheads) are labelled with *TgBAC(pdgfrβ:GFP)* and vessels (red) are labelled with *Tg(kdrl:mCherry)*. There are significantly fewer brain pericytes (C) and reduced pericyte density (D) in nkx3.1$^{-/-}$ mutant as compared to nkx3.1$^{+/-}$ controls. Statistics used a Student's t-test. (n=9 wildtypes, 6 mutants). Scale bar is 50 µm. The data underlying this figure can be found in S3 Table.
