# Supplementary material for: The development of brain pericytes requires expression of the transcription factor nkx3.1 in intermediate precursors
Source: PLoS Biol. 2024 Apr 29;22(4):e3002590. doi: 10.1371/journal.pbio.3002590 (PMC11081496; doi:10.1371/journal.pbio.3002590)
Supplement: S6 Fig — (A) Overlay of UMAP projections from 2 biologically independent scRNAseq samples (1, red; 2, blue) showing cells with similar distributions from both samples. (B) Stacked barplot showing the proportion of cells in each cluster deriving from the first sample [1] or second sample [2] as normalized as a percentage to the total number of cells from each sample. (Right) Table of proportions of the different cell types in each sample. (PDF) [file pbio.3002590.s012.pdf]

A

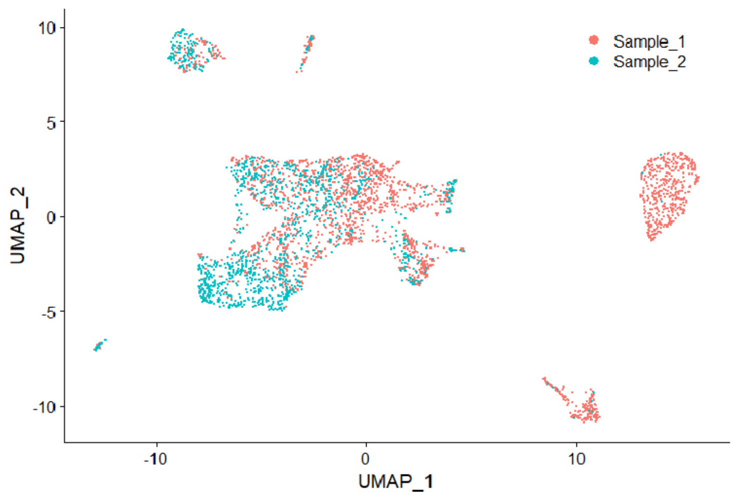

B

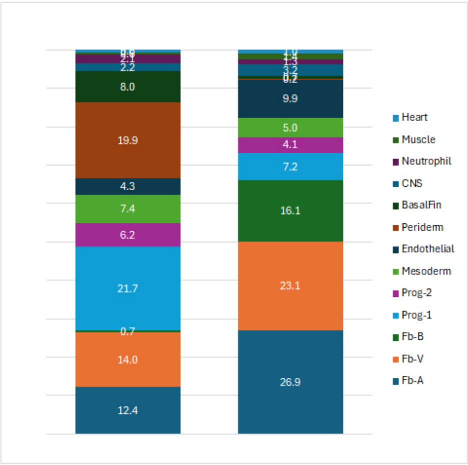

Cluster	Sample_1	Sample_2
Fb-A	12.4	26.9
Fb-V	14.0	23.1
Fb-B	0.7	16.1
Prog-1	21.7	7.2
Prog-2	6.2	4.1
Mesoderm	7.4	5.0
Endothelial	4.3	9.9
Periderm	19.9	0.2
BasalFin	8.0	0.7
CNS	2.2	3.2
Neutrophil	2.1	1.3
Muscle	0.6	1.4
Heart	0.6	1.0

S6 Fig: Analysis of proportions and batch effects analysis of scRNAseq

(A) Overlay of UMAP projections from two biologically independent scRNAseq samples (1, red, 2, blue) showing cells with similar distributions from both samples. (B) Stacked barplot showing the proportion of cells in each cluster deriving from the first sample (1) or second sample (2) as normalized as a percentage to the total number of cells from each sample. (Right) Table of proportions of the different cell types in each sample.
