# Supplementary figures and images for: The development of brain pericytes requires expression of the transcription factor nkx3.1 in intermediate precursors
Source: PLoS Biol. 2024 Apr 29;22(4):e3002590. doi: 10.1371/journal.pbio.3002590 (PMC11081496; doi:10.1371/journal.pbio.3002590)

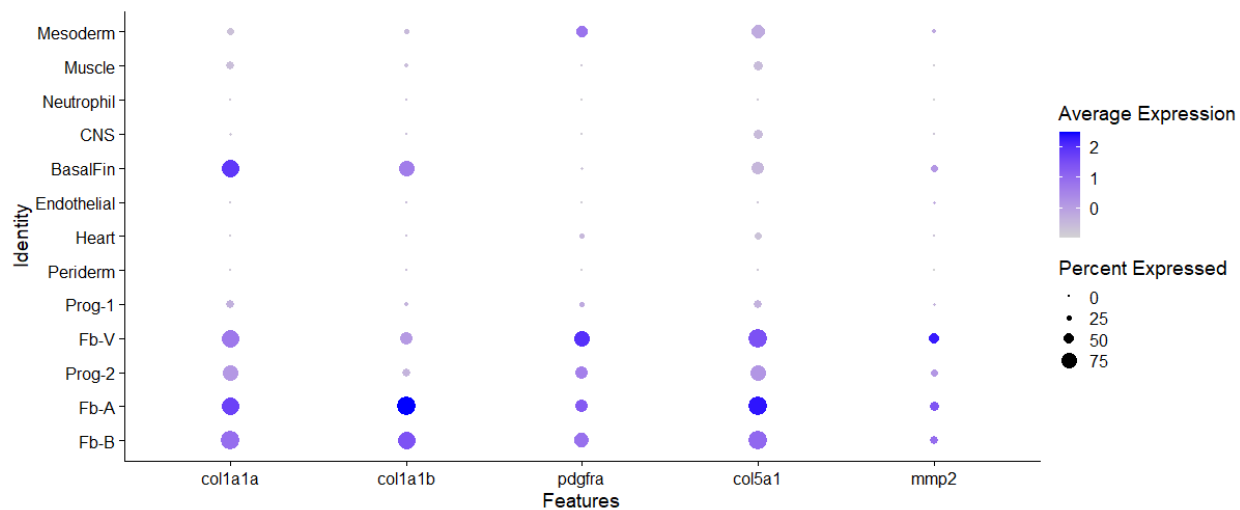

**S7 Fig: Dotplot showing expression of common fibroblast markers in Progenitor-2 and Fb clusters**

Supplement: S7 Fig — (PDF) [file pbio.3002590.s013.pdf]

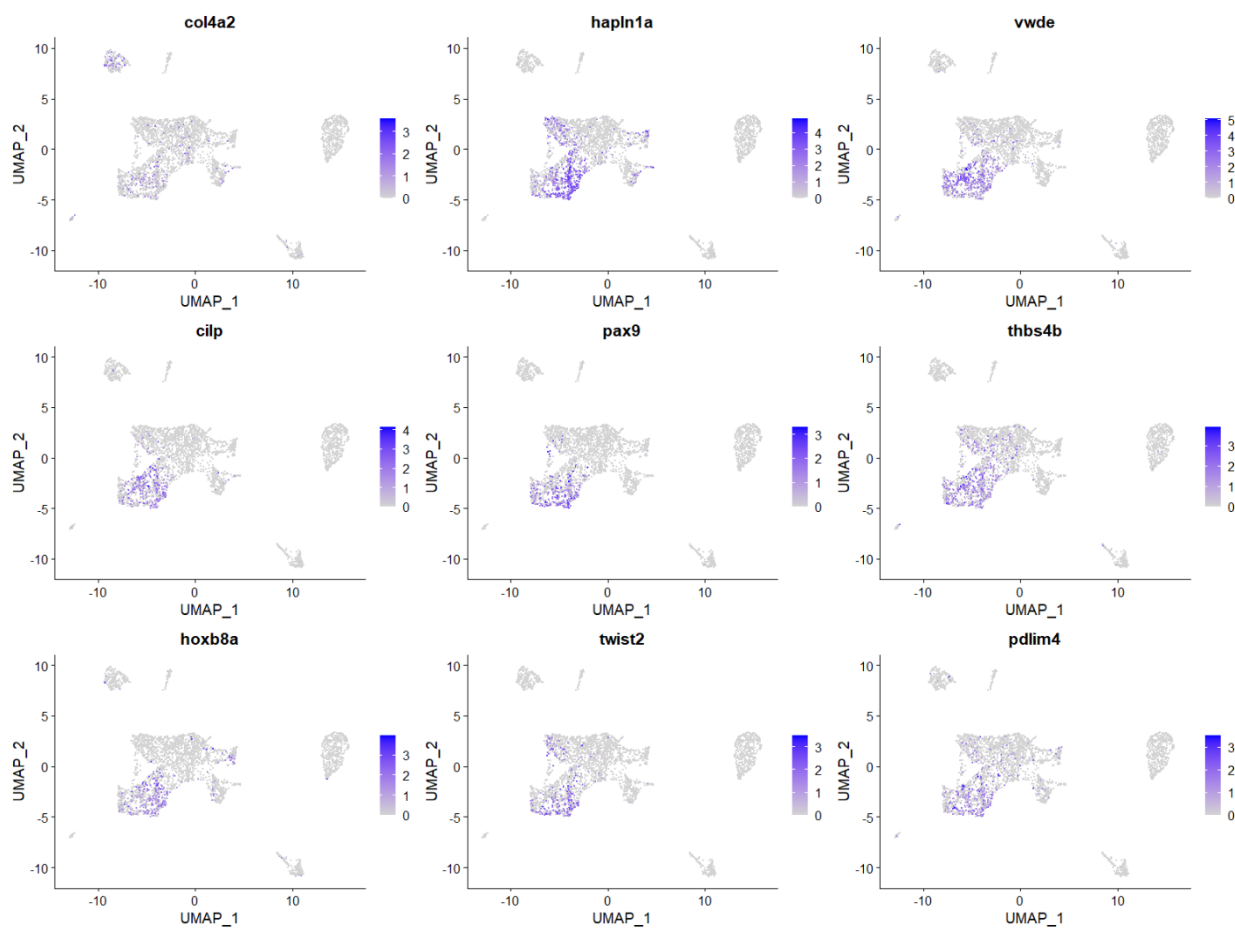

**Fig. S9: Featureplots of genes enriched in the Fb-A scRNAseq cluster**

Supplement: S9 Fig — (PDF) [file pbio.3002590.s015.pdf]

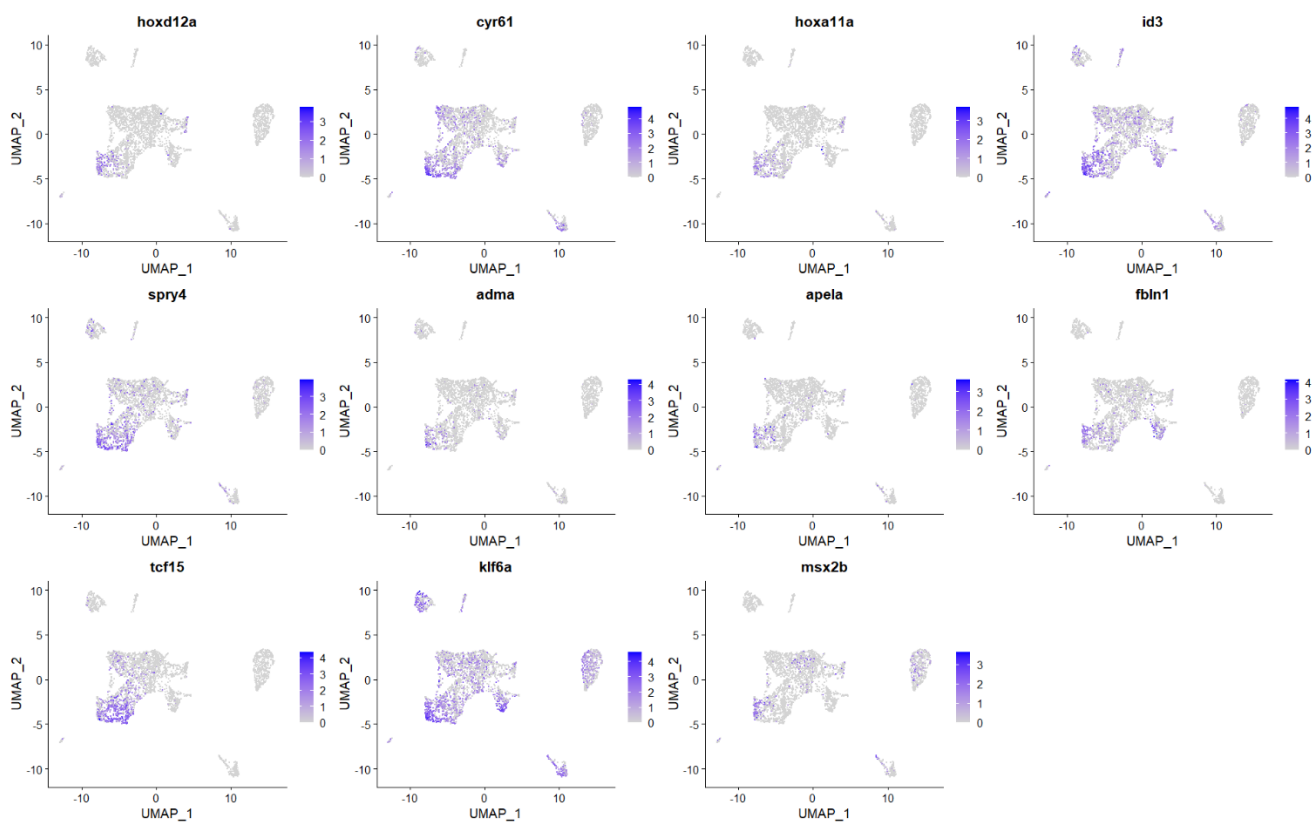

**S10 Fig: Featureplots of genes enriched in the Fb-B scRNAseq cluster**

Supplement: S10 Fig — (PDF) [file pbio.3002590.s016.pdf]

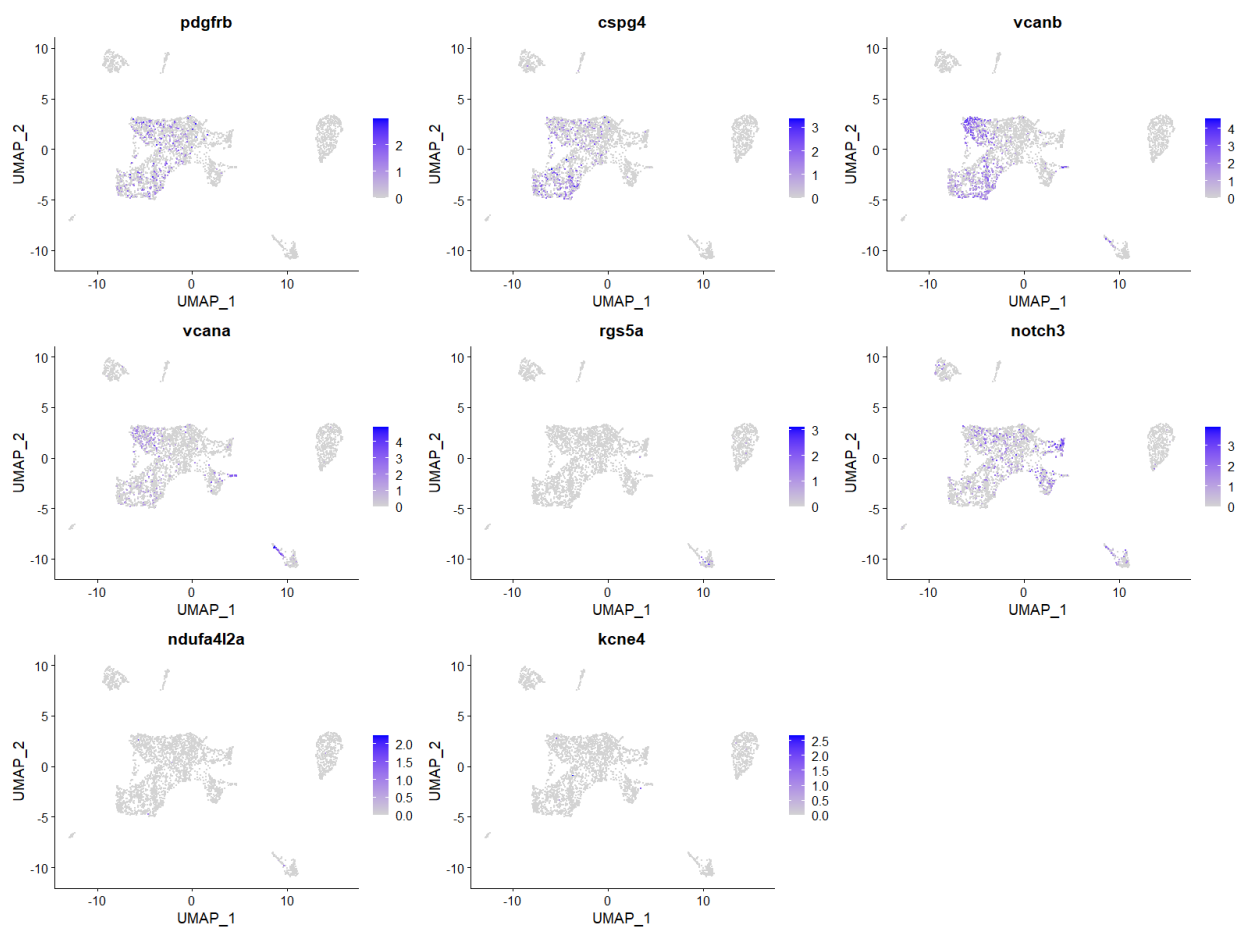

**S11 Fig: Featureplots of canonical pericyte markers in the nkx3.1-positive cell scRNAseq data.**

Supplement: S11 Fig — (PDF) [file pbio.3002590.s017.pdf]
