# Supplementary material for: The development of brain pericytes requires expression of the transcription factor nkx3.1 in intermediate precursors
Source: PLoS Biol. 2024 Apr 29;22(4):e3002590. doi: 10.1371/journal.pbio.3002590 (PMC11081496; doi:10.1371/journal.pbio.3002590)
Supplement: S8 Fig — (PDF) [file pbio.3002590.s014.pdf]

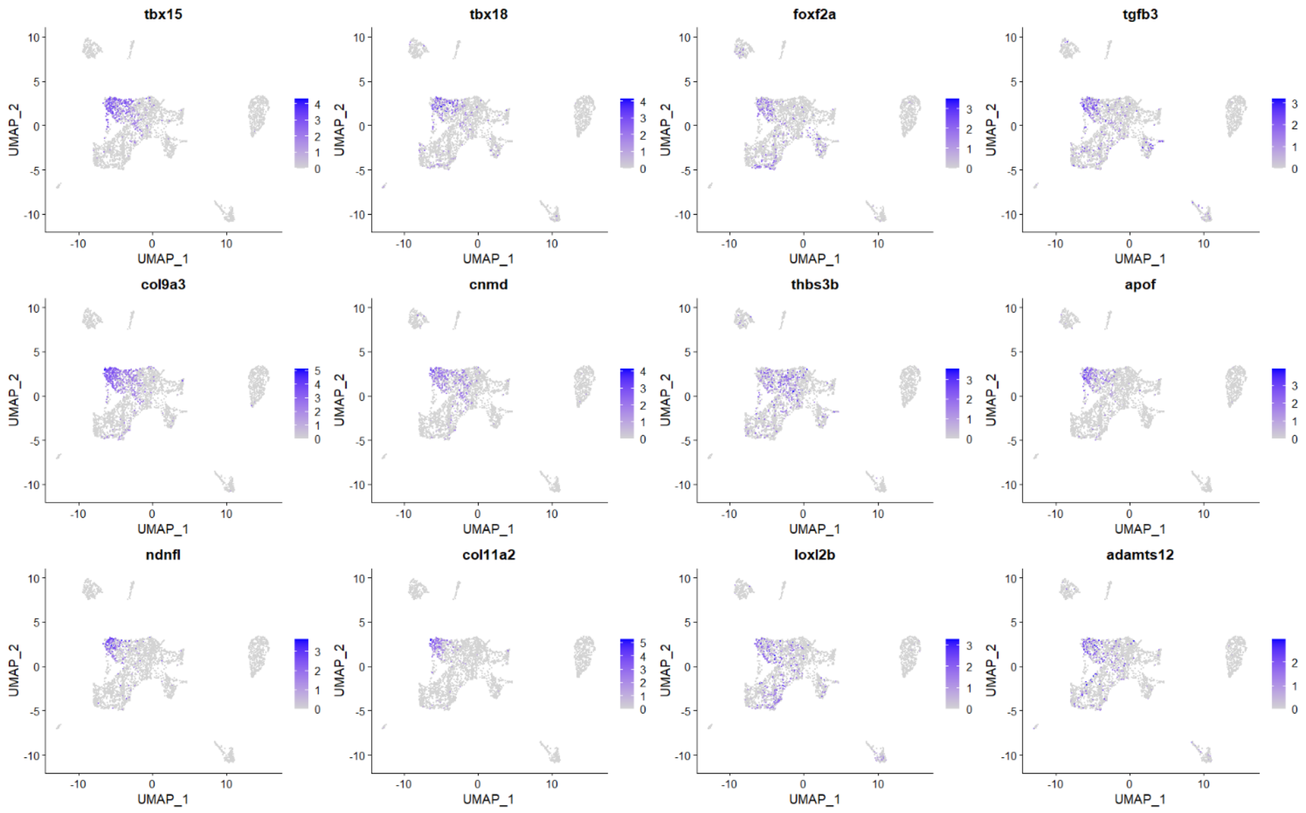

S8 Fig: Featureplots of genes enriched in the Fb-V scRNAseq cluster (fibroblast-like pericyte precursors)
