# Supplementary material for: The development of brain pericytes requires expression of the transcription factor nkx3.1 in intermediate precursors
Source: PLoS Biol. 2024 Apr 29;22(4):e3002590. doi: 10.1371/journal.pbio.3002590 (PMC11081496; doi:10.1371/journal.pbio.3002590)
Supplement: S12 Fig — (A) Dorsal view of the posterior head region of 16 hpf embryo showing expression overlap (white bracket) between cxcl12b (green) and nkx3.1 (red). (B) Lateral view of the posterior head region of 24 hpf embryo showing expression overlap (white bracket) between cxcl12b and nkx3.1. A-Anterior, P-Posterior, D-Dorsal, V-Ventral. Scale bar is 50 μm. (PDF) [file pbio.3002590.s018.pdf]

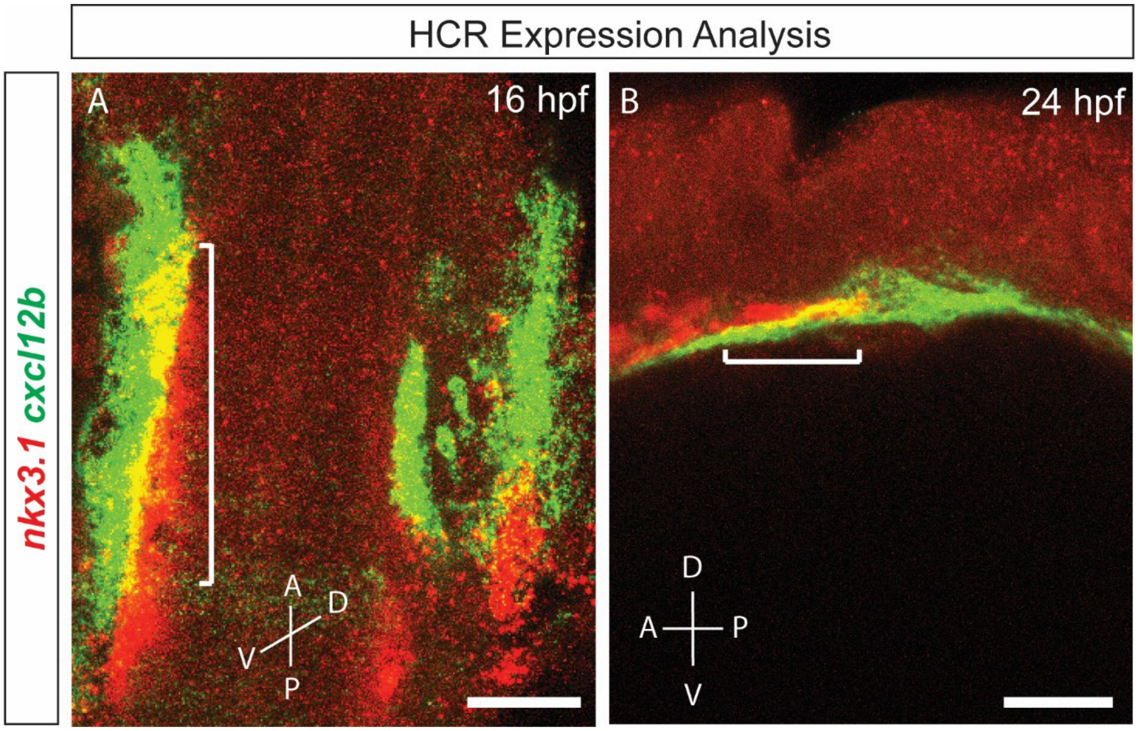

S12 Fig: Expression analysis of *nkx3.1* and *cxcl12b* using HCR. (A) Dorsal view of the posterior head region of 16 hpf embryo showing expression overlap (white bracket) between *cxcl12b* (green) and *nkx3.1* (red). (B) Lateral view of the posterior head region of 24 hpf embryo showing expression overlap (white bracket) between *cxcl12b* and *nkx3.1*. A-Anterior, P-Posterior, D-Dorsal, V-Ventral. Scale bar is 50 μm.
