# Supplementary material for: The development of brain pericytes requires expression of the transcription factor nkx3.1 in intermediate precursors
Source: PLoS Biol. 2024 Apr 29;22(4):e3002590. doi: 10.1371/journal.pbio.3002590 (PMC11081496; doi:10.1371/journal.pbio.3002590)
Supplement: S13 Fig — (A-C) Dorsal views of embryonic brain of uninjected control (A) and cxcl12b mRNA injected (B) embryos showing an increase in brain pericyte number. (C) Quantitation of brain pericytes (n = 5 wild-type and 9 injected embryos). (D-F) Dorsal views of embryonic brain of uninjected nkx3.1−/− (D) and cxcl12b mRNA injected nkx3.1−/− embryos (E) showing an increase in brain pericyte number due to cxcl12b mRNA injection (F, n = 10 uninjected and 10 injected mutants). Pericytes (green, arrowheads) are labelled with TgBAC(pdgfrβ:GFP) and vessels (red) are labelled with Tg(kdrl:mCherry). (G) Hemorrhage rates were unchanged in mutants at 48 hpf (N = 3 with 141 uninjected at 167 injected mutants analyzed). Statistics used the Student t test. Scale bar is 50 μm. The data underlying this figure can be found in S3 Table. (PDF) [file pbio.3002590.s019.pdf]

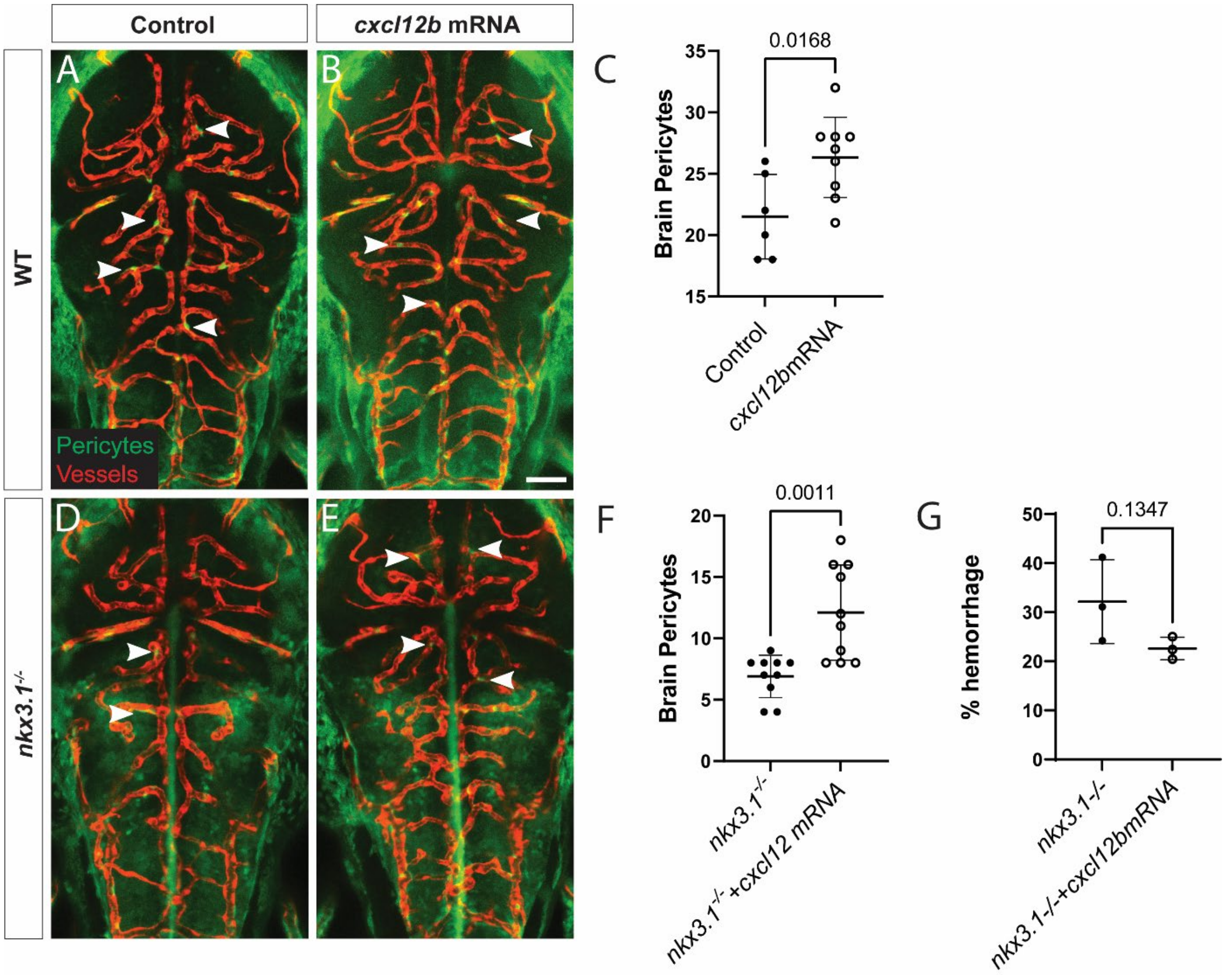

S13 Fig: *cxcl12b* mRNA injection increases pericyte numbers at 75 hpf. (A-C) Dorsal views of embryonic brain of uninjected control (A) and *cxcl12b* mRNA injected (B) embryos showing an increase in brain pericyte number (C) Quantitation of brain pericytes (n= 5 wildtype and 9 injected embryos). (D-F) Dorsal views of embryonic brain of uninjected *nkx3.1*$^{-/-}$ (D) and *cxcl12b* mRNA injected *nkx3.1*$^{-/-}$ embryos (E) showing an increase in brain pericyte number due to cxcl12b mRNA injection (F, n=10 uninjected and 10 injected mutants). Pericytes (green, arrowheads) are labelled with *TgBAC(pdgfrβ:GFP)* and vessels (red) are labelled with *Tg(kdrl:mCherry)*. (G) Hemorrhage rates were unchanged in mutants at 48 hpf (N=3 with 141 uninjected at 167 injected mutants analyzed). Statistics used the Students T-test. Scale bar is 50 µm. The data underlying this figure can be found in S3 Table.
