# Supplementary material for: The development of brain pericytes requires expression of the transcription factor nkx3.1 in intermediate precursors
Source: PLoS Biol. 2024 Apr 29;22(4):e3002590. doi: 10.1371/journal.pbio.3002590 (PMC11081496; doi:10.1371/journal.pbio.3002590)
Supplement: S14 Fig — Dorsal view of the ventral head region of 36 hpf embryo showing no expression overlap between cxcl12b (green) and cxcr4a (red). A-Anterior, P-Posterior, Scale bar is 20 μm. (PDF) [file pbio.3002590.s020.pdf]

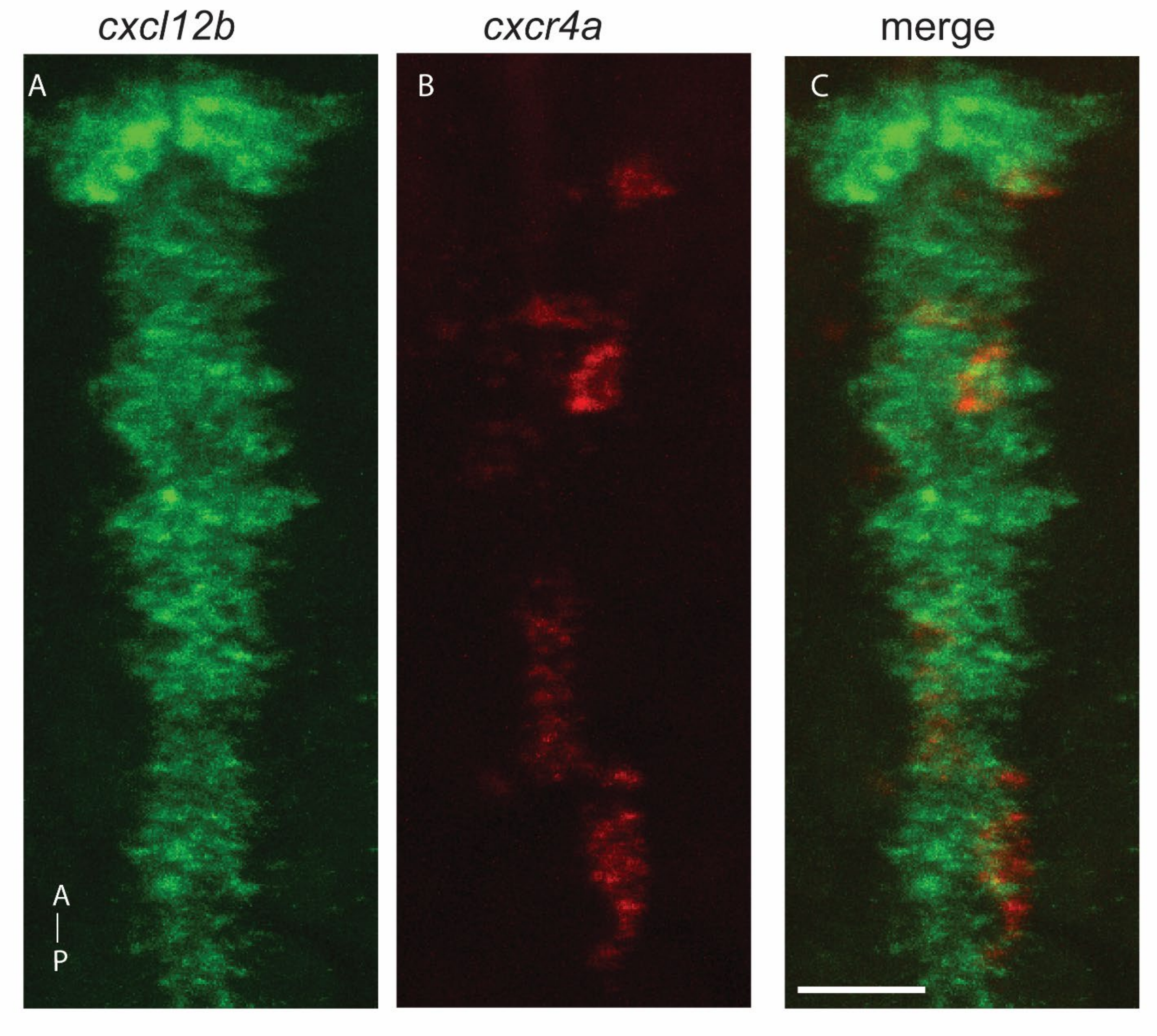

S14 Fig: No overlap between *cxcl12b* and cxcr4a mRNA at 36 hpf

Dorsal view of the ventral head region of 36 hpf embryo showing no expression overlap between *cxcl12b* (green) and *cxcr4a* (red). A-Anterior, P-Posterior, Scale bar is 20 μm.
