# Supplementary material for: The development of brain pericytes requires expression of the transcription factor nkx3.1 in intermediate precursors
Source: PLoS Biol. 2024 Apr 29;22(4):e3002590. doi: 10.1371/journal.pbio.3002590 (PMC11081496; doi:10.1371/journal.pbio.3002590)
Supplement: S15 Fig — Output of searches for early pericyte markers showing the pericyte cluster expression at 24 hpf–48 hpf of the indicated genes (red box). (PDF) [file pbio.3002590.s021.pdf]

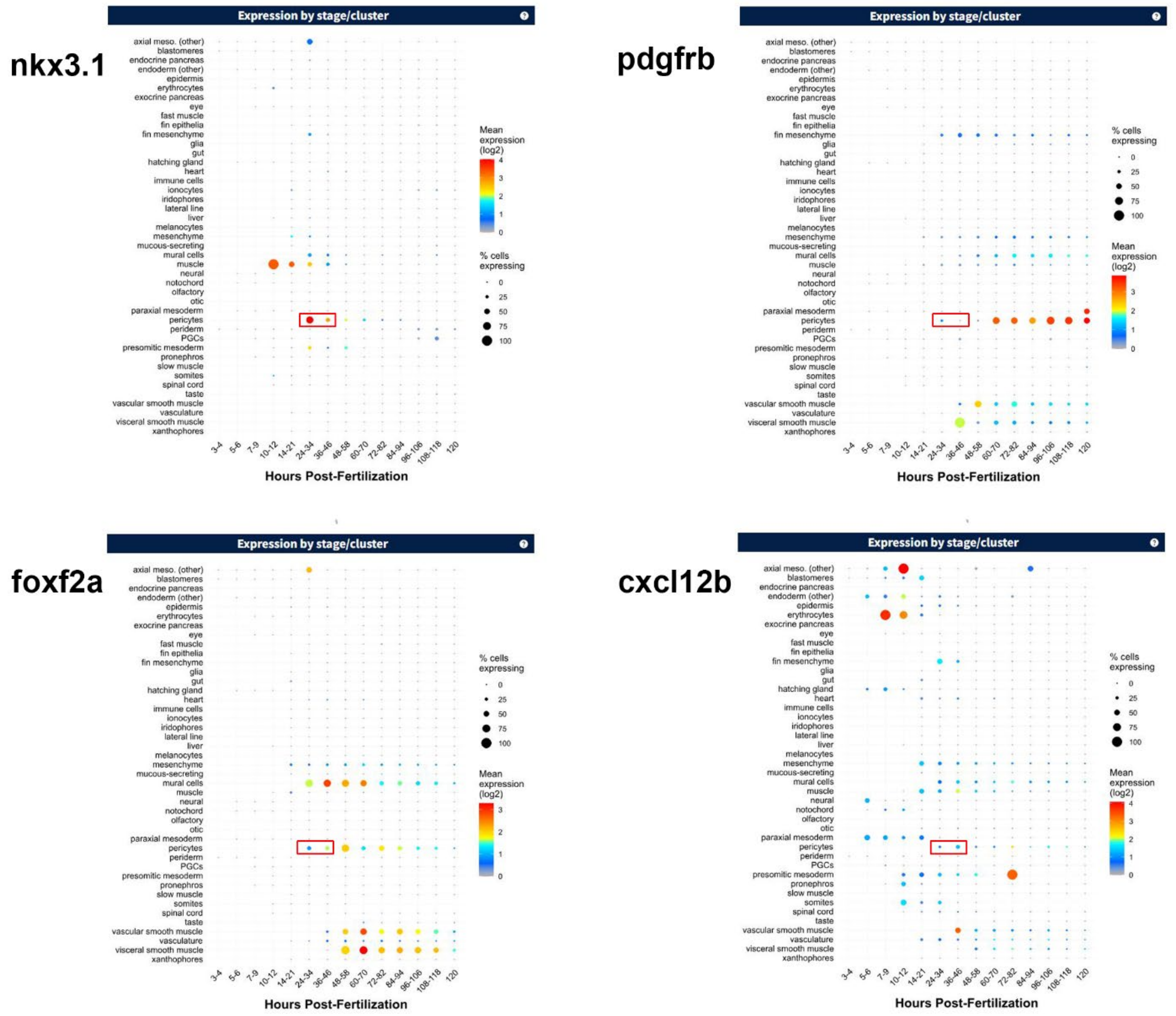

S15 Fig: Expression of pericyte precursor genes in the Daniocell database Output of searches for early pericyte markers showing the pericyte cluster expression at 24 hpf – 48 hpf of the indicated genes (red box).
