# Supplementary material for: The development of brain pericytes requires expression of the transcription factor nkx3.1 in intermediate precursors
Source: PLoS Biol. 2024 Apr 29;22(4):e3002590. doi: 10.1371/journal.pbio.3002590 (PMC11081496; doi:10.1371/journal.pbio.3002590)
Supplement: S1 Data — (DOCX) [file pbio.3002590.s022.docx]

## Performing velocity analysis using Seurat and R implementation of velocyto

## Reference guide : https://github.com/satijalab/seurat-wrappers/blob/master/docs/velocity.md

##load required libraries

library(Seurat)

library(velocyto.R)

library(SeuratWrappers)

## reading barcodes of a loom file

x<-read.table("colnames.txt", sep="\t", header=FALSE)

avector<-x[['V1']]

### Load filtered Seurat object and read loom file

seurat_object <- readRDS('seuratObject.rds')

ldat <- ReadVelocity(file = "file.loom")

### Subset cells or barcodes of a loom file to map cells in a filtered seurat object

for (i in names(x = ldat)) {

### Store assay in a new variable

assay <- ldat[[i]]

colnames(assay)<-avector

### Subset to filtered cells in Seurat object

assay <- assay[,colnames(seurat_object)]

### Add assay to Seurat object

seurat_object[[i]] <- CreateAssayObject(counts = assay)

}

ident.colors<-c(`0` ='#ff68a1', `1` ='#1FA195', `2` ='#B95FBB',`3` ='#CCB1F1',`4` ='#aeadb3',`5` ='#F68282', `6` ='#00A9FF', `7` ='#D4D915', `8` ='#ff61cc',`9` ='#28CECA',`10` ='#31C53F',`11` = "#CD9600",'12' ='#aeadb3')

#names(x = ident.colors) <- levels(x = seurat_object)

cell.colors <- ident.colors[Idents(object = seurat_object)]

names(x = cell.colors) <- colnames(x = seurat_object)

### RUNVELOCITY

seurat_object<- RunVelocity(object = seurat_object, deltaT = 1, kCells = 25, fit.quantile = 0.02)

#seurat_object<- RunVelocity(object = seurat_object, assay="spliced", deltaT = 1, kCells = 25, fit.quantile = 0.02)

show.velocity.on.embedding.cor(emb = Embeddings(object = seurat_object, reduction = "umap"), vel = Tool(object = seurat_object,

slot = "RunVelocity"), n = 200, scale = "sqrt", cell.colors = ac(x = cell.colors, alpha = 0.5),

cex = 0.8, arrow.scale = 3, show.grid.flow = TRUE, min.grid.cell.mass = 0.5, grid.n = 40, arrow.lwd = 1,

do.par = FALSE, cell.border.alpha = 0.1)
