# Supplementary material for: The development of brain pericytes requires expression of the transcription factor nkx3.1 in intermediate precursors
Source: PLoS Biol. 2024 Apr 29;22(4):e3002590. doi: 10.1371/journal.pbio.3002590 (PMC11081496; doi:10.1371/journal.pbio.3002590)
Supplement: S2 Data — (DOCX) [file pbio.3002590.s023.docx]

library(dplyr)

library(Seurat)

library(patchwork)

library(hdf5r)

library(ggplot2)

#set working directory

# Load data

# Initialize the Seurat object with the raw (non-normalized data).

nkx31 <- CreateSeuratObject(counts = nkx31.data, project = "30hpf_pericyte", min.cells = 3, min.features = 200)

nkx31

# QC stats

nkx31[["percent.mt"]] <- PercentageFeatureSet(nkx31, pattern = "^mt-")

# Visualize QC metrics as a violin plot

VlnPlot(nkx31, features = c("nFeature_RNA", "nCount_RNA", "percent.mt"), ncol = 3)

#QC- removal of bad cells

nkx31 <- subset(nkx31, subset = nFeature_RNA > 200 & nFeature_RNA < 2500 & percent.mt < 5)

#Normalie the data

nkx31 <- NormalizeData(nkx31)

#Find variable features

nkx31 <- FindVariableFeatures(nkx31, selection.method = "vst", nfeatures = 2000)

#Scaling the data

all.genes <- rownames(nkx31)

nkx31 <- ScaleData(nkx31, features = all.genes)

#linear dimensional reduction

nkx31 <- RunPCA(nkx31, features = VariableFeatures(object = nkx31))

DimPlot(nkx31, reduction = "pca")

DimHeatmap(nkx31, dims = 1, cells = 500, balanced = TRUE)

DimHeatmap(nkx31, dims = 1:15, cells = 500, balanced = TRUE)

#Determining dimensionality

nkx31 <- JackStraw(nkx31, num.replicate = 100)

nkx31 <- ScoreJackStraw(nkx31, dims = 1:20)

JackStrawPlot(nkx31, dims = 1:15)

#Clustering

nkx31 <- FindNeighbors(nkx31, dims = 1:15)

nkx31 <- FindClusters(nkx31, resolution = 0.6)

#Run UMAP

nkx31 <- RunUMAP(nkx31, dims = 1:15)

#DimPlot(nkx31, reduction = "umap")

DimPlot(nkx31, reduction = "umap", label = TRUE, label.size = 6, pt.size = 1) + NoLegend()
