# Supplementary material for: The development of brain pericytes requires expression of the transcription factor nkx3.1 in intermediate precursors
Source: PLoS Biol. 2024 Apr 29;22(4):e3002590. doi: 10.1371/journal.pbio.3002590 (PMC11081496; doi:10.1371/journal.pbio.3002590)
Supplement: Attachment — Submitted filename: Nkx3.1 reply to reviewers rev.pdf [file pbio.3002590.s024.pdf]

We thank the reviewers for their appreciation of our study and for the careful comments that have led to significant improvements in our manuscript. We have replied to each point below and have attached a marked-up copy of the manuscript with major additions/text changes noted.

Please note that the pdf generated by the submission system is low resolution and has exaggerated the colors of the figures. If possible, the reviewers should look at the original tif's that were uploaded to the system, not the pdf.

Reviewer #1: Summary:

In this manuscript, Ahuja et al. have sought to define a precursor cell population that differentiates into brain pericytes and identify genetic programs responsible for this process. This is an outstanding question in cerebrovascular biology; thus, this study provides novel and important biological insights in this field. In this manuscript, the authors specifically focused on investigating an early embryonic cell population that expresses the transcription factor Nkx3.1 and determining how this cell lineage differentiates into brain pericytes. This study has been carried out by combining novel molecular genetic zebrafish tools with bulk and single-cell transcriptomic analyses through LOF, GOF, global or cell type-specific rescue experiments, and cell lineage tracing. A variety of the modern and elegant techniques employed in this manuscript are impressive and make this study stand out.

Overall, this study is very well-crafted, nicely presented, and well described. Experiments were carefully designed and executed, making the findings of this manuscript solid and rigorous in most parts. Moreover, this study seeks to fill an important gap in our scientific knowledge by revealing the developmental trajectories of brain pericytes. This is a crucial next step in both developmental neurobiology and vascular biology fields, and their findings will provide valuable insights into brain pericyte development. Despite the significance of this study, however, I have several critical points that will need to be addressed by the authors to improve the current format of the manuscript.

Major Comments:

1-1) Most of the major conclusions the authors have drawn in this manuscript are based on their quantifications for the number of brain pericytes, which were conducted at the single time point 75 hpf. Since the authors showed disrupted patterning of brain vascular networks in some, or most, loss- and gain-of-function genetic experiments and after pharmacological treatments, the reduced number of brain pericytes could be due to the mis-patterning and/or reduced complexity of brain vascular networks. Thus, it would be more appropriate to present these quantification results by pericyte density per vessel length rather than absolute pericyte numbers that may rely on the extent of vascular network elaboration. Presenting both quantifications will be more informative and strengthen the findings.

This is a thoughtful point that all 3 reviewers queried. During the submission/revision of this manuscript our lab has finalized and published software that can calculate endothelial parameters relevant to this study, most particularly vessel diameter and vessel network length (Vessel metrics: DOI: 10.1016/j.mvr.2023.104610). We have used it in this revision to quantitate pericyte density by measuring vessel network length of the central arteries of the hindbrain, and pericyte number along these vessels. Pericyte density graphs are now present in Figure 2, 4, 5, S4, S5 showing that pericyte density is decreased in mutants, while pericyte density is increased in gain of function. This is the same trend as pericyte numbers. We find that network length doesn't change in any of the manipulations suggesting a pericyte-specific phenotype. Vessel diameter measurements have been added to Figure 2.

1-2) Related to my major point #1, phenotypic assessments at a single-time point raises a concern about data interpretation of observed phenotypes. For example, a MZ nkx3.1 mutant embryo at 52 hpf (Fig. 2B) displays peri-cardiac edema, bigger yolk sac, and brain ventricular enlargement (hydrocephalus) compared to the heterozygous animal (Fig. 2A), raising a concern that there is a significant developmental delay in the mutant partly due to impaired blood circulation resulting from peri-cardiac edema and weakened heartbeats. Similarly, 75 hpf larvae (Fig. 2D and 2E) showed a substantial difference in their brain size (much smaller brain in the mutant). Again, this brain size difference raises a concern about the interpretation of the authors' quantification data because significantly mismatched developmental stages were potentially used for quantifications and phenotypic comparisons. This is a critical point because pericyte reduction in MZ nkx3.1 mutants is much milder than the previously reported phenotypes observed in pdgfrb and notch3 zebrafish mutant larvae, which exhibit a near-complete or severe loss of brain pericytes (Ando et al., Dev. Biol., 2021; Wang et al., Development, 2014). The bottom line is that phenotypic analysis conducted at only a single time point makes the conclusions weak in some contexts due to the concerns I raised above. The authors are recommended to analyze phenotypes at additional time point(s) to address this type of concerns, especially for brain pericyte number quantifications (Fig. 2F).

This is also a good point. Just to clarify, although there is occasional hydrocephalus and edema, this is not present in all mutants, and is mild when present. nkx3.1 mutants are homozygous viable as adults, although live a shortened lifespan as compared to normal fish (~1 year as opposed to ~2 years). The adult homozygous viable state allows us to make MZ mutants. We have added 5 dpf quantification to the revision (S5 Fig). Pericyte number is reduced by ~25% and density by 20% at 5 dpf, similar to the phenotype at 3 dpf. Network length is unchanged between mutants and wildtype. This indicates that there is a persistent phenotype at larval stages in nkx3.1 mutants, and that the phenotype is not a result of developmental delay.

1-3) Related to the authors' proposed model, if Nkx3.1 acts upstream of Cxcl12-Cxcr4 signaling that may subsequently regulate the Pdgfrb signaling pathway, how do the authors explain the much milder pericyte-loss phenotypes observed in MZ nkx3.1 mutants and after AMD3100 treatment (Cxcr4 inhibition) compared to pdgfrb mutants? This point and apparent discrepancy should be discussed.

When dosing inhibitors in our laboratory, we titrate the dose to a level that gives the maximal phenotype with minimal off-target effects (i.e. so that the drug gives no hydrocephalus, edema or embryo developmental defects at 3 dpf). It is likely that the dose of AMD3100 doesn’t completely inhibit Cxcr4.

Since many parallel pathways contribute to development, it is not unreasonable that nkx3.1 has an overlapping but different phenotype from pdgfrb LOF as it could be involved in some, but not all aspects of specification, migration, proliferation and differentiation of pericytes. As we show, there are at least 3 transcription factors that are present in the same population of cells, potentially with overlapping roles (foxf2, tbx18, tbx15, nkx3.2). We would suggest that double loss of function might be needed to completely ablate pericyte due to partial redundancy.

1-4) Loss of cxcl12b expression in nkx3.1 mutant embryos is drastic and convincing (Fig. 4A, 4B), but it is not clear how this secreted ligand controls pericyte differentiation process in Nkx3.1-positive precursors. Given the crucial role of this chemokine signaling in vSMC recruitment in the dorsal aorta of the zebrafish trunk (Stratman et al., Commun. Biol., 2020) combined with endothelial cxcl12b expression shown in Fig. 3N, it is possible that endothelial cells-derived Cxcl12b contributes to brain pericyte recruitment. Although rescue experiments are elegantly performed in Fig. 5, this part of the study is still under-developed partly due to the limited spatial

information on cxcl12b and cxcr4 expression and their cellular sources. It would be much more informative if the authors would perform HCR co-expression analysis of cxcl12b and cxcr4a, and/or their individual expression analysis in a vascular endothelial reporter background and/or together with a nkx3.1 probe. These data will likely strengthen this part of the study and provide data to suggest a better cellular and signaling model.

We added new HCR data (S13 Fig) to show that cxcl12b and cxcr4a show no overlap in expression at this stage. Further, looking at the scRNAseq data for expression of cxcl12b, even though Figure 3N has a few blue dots in the endothelial cell cluster, Seurat does not find it as a cluster 5 marker (endothelial cells), while cxcl12b is a marker of multiple fibroblast clusters. Thus, between the HCR and the scRNAseq, we have no evidence for autocrine signaling in endothelial cells. Yes, there must be additional paracrine signalling from pericytes to endothelial cells that would explain the differentiation role (known signals would include Pdgf-Pdgfrb and Ang-Tie). We have shown nkx3.1 and cxcl12b co-expression at 16 and 24 hpf but we are not able to do the nkx3.1 and Kdrl co-expression analysis at 36 hpf, because nkx3.1 mRNA is not expressed. However, the overlap of nkx3.1 with cxcl12b, and the non-overlap of cxcl12b with cxcr4 (endothelial cells) implies that nkx3.1 is not expressed in endothelial cells. Our sequencing experiments also suggest there is no overlap.

1-5) Are the images that showed expression overlaps of two genes (Fig. 3E-M and Fig. S10) presented as z-stack projection or single-plane images? Expression overlap should be shown on a single-plane frame. Please also clarify this information in the legend.

We have added the information to the legend of Figure 3. These are HCR fluorescent in situ hybridization. Images shown are sub-stacks of a few planes in the region of the forming basilar artery and precursor area.

1-6) No descriptions of the quantification means are provided in the Methods section. Please provide detailed descriptions on how all the quantifications presented in this manuscript were performed.

The methods have been updated to describe how the pericytes were counted from the imaging data and how the pericyte density was calculated. We apologize for this omission.

Minor Comments:

1-7) Please provide explanations on the specificity of TgBAC(nkx3.1:Gal4)ca101 expression patterns in the developing brain as the cited original paper did not focus on the brain.

In Figure 1 we show complete overlap of the pdgfrβ transgene and nkx3.1 transgene in the brain of developing embryos. The expression of the two transgenics are very similar inside the head and brain. We did not find evidence for non-pdgfrβ-expressing perivascular fibroblasts in the brain.

1-8) In Fig. 1A-C panels, please add the indication of A-P and D-V axes, similar to Fig. S10.

We have updated figure 1.

1-9) In Fig. 2A-C legends, please include animal numbers for the nkx3.1+/- genotype.

We have added these numbers.

1-10) In Fig. 5F graph, please add the P-value information.

We have done this. It is 0.1776

Reviewer #2: General comments:

The authors identified the transcription factor Nkx3.1 as regulating development of mature pericytes from precursors. By lineage tracing experiments, they identified that nkx3.1 is expressed in precursor cell populations which later developed into pericytes. By conducting a rescue experiment, the authors showed that Cxcl12b acts downstream of Nk3.1. Overall, the study used a combination of genetics and transcriptomics and proposed a mechanism where early pericyte differentiation is regulated by Nk3.1-cxcl12b signalling.

While several molecular pathways are known to be involved in pericyte development (pdgfrb, notch) there is a lack of understanding of early transcriptional regulators and differentiation. This study would identify an important transcription factor in pericyte development and at least one key target in cxcl12b. This finding is likely to be well received by the vascular biology field and open up new understanding in pericyte development.

In my opinion, this is an extensive study and presents novel findings in pericyte development. However, there are still several issues with data and interpretation. See below.

Major:

2-1. The authors mention that nkx3.1 MZ mutants display mispatterned vessels. This is a vague term that needs to be addressed. Furthermore, there is no quantification and the representative images do not show an obvious defect. The authors need to carefully examine and quantify the vascular phenotypes such as volume, coverage and branching. Furthermore, all pericyte quantifications need to be adjusted to be expresses relative to vascular coverage in order to rule out the possibility that changes in pericyte numbers are due to altered vasculature overall.

This is similar to the query 1-1 above. We have added pericyte density, a measure that incorporates total vessel network length and pericyte number, to the figures. The central artery (hindbrain) network length was quantified and added to the supplement. Mispatterned was a poor choice of words, and none of our tools could pick up a quantitative difference in patterning. We therefore have omitted this word from the manuscript.

2-2. Does nkx3.1 generally impact all early mesenchymal fibroblasts development? Or just the precursors of the pericytes? Quantification of pdgfrb low expressing cells in the mutants or using other fibroblast markers would give more confidence in the specificity of the phenotype to pericytes.

We and others (Rajan et al., https://doi.org/10.1101/2023.04.18.537403) have shown through single cell sequencing that nkx3.1 lineage gives rise to diverse cell types including traditional trunk fibroblasts from the sclerotome. Thus, multiple cell types would likely be affected by nkx3.1 mutation. Indeed, others have published that trunk fibroblasts are affected in fish when nkx3.1-expressing cells are ablated (Rajan, PLOS Genetics, 2020). We are the first to publish the mutant phenotype and to examine the phenotype in the brain. We don’t see pdgfrb-low cells in the brain that aren’t co-expressing nkx3.1 or vice versa. There is 100% overlap.

2-3. The authors should give further details about the gross morphological defects displayed in MZ nkx3.1 mutants. Do these animals survive the larval stages? Do they have delayed development? Is there a restricted blood supply to the brain or other potentially affected tissues that in turn cause a general reduction in tissue size, vessel formation and pericyte development?

Similar to query 1-2 above, we note that nkx3.1 mutants are homozygous viable as adults, and that embryonic phenotypes (other than a reduction in pericyte numbers), are transient, rare, and mild. We do not think that the phenotype is due to developmental delay and now show a quantitatively similar phenotype at 5 dpf (S5 Fig.).

2-4. The authors claim that hemorrhage in nkx3.1 MZ mutants occurs as a result of pericyte loss. Ando et al 2022 showed that pdgfrb mutants, which lack most brain pericytes except those on the metencephalic artery, do not display hemorrhage until juvenile stages. Considering that pericyte loss in pdgfrb mutants is more severe than in nkx3.1 MZ mutants, the authors claim seems very unlikely to be correct. The nature and cause of the haemorhages should be characterised more carefully. Do the blood vessels rupture? Are junctions intact? Are fibroblasts likely to play a role as well as pericytes? The authors need to clarify this point, preferably with additional data.

This is an interesting point, as the reviewer contrasts different mutants with respect to pericyte reduction and hemorrhage arguing that the lack of pericytes per se cannot be the cause of hemorrhage given the pdgfrb mutant. The reference for hemorrhage and pericyte reduction is a mouse notch 3 knockout, and we have previously found both phenotypes in foxc1 mutants (Whitesell et al, DB 2019), and foxf2 mutants (Ryu, PNAS 2022). Foxf2 is co-expressed in the Fb-V cluster. We agree that although these two phenotypes frequently co-occur, the lack of pericytes may not be causal. We have softened wording to ‘phenotypes associated with pericyte reduction’ and referenced these three articles.

2-5. nkx3.1 mutants seem likely to have defects in pericyte development in other areas of the embryo. Perhaps examination of the trunk vasculature and pericytes would strengthen the hypothesis of nkx3.1's specific role in brain pericyte development.

Rajan et al., (PlosGenetics 2020) use the nkx3.1:gal4;uas:ntr-mCherry to show effects on trunk fibroblasts. Since trunk and brain pericytes are derived from different embryonic populations, our work on the role of nkx3.1 in brain pericytes is unique. But yes, nkx3.1 is involved in pericyte development in different areas of the embryo.

2-6. Does the cxcl12b injection rescue formation of early brain hemorrhage that occurs in nkx3.1 MZ mutants? If so, this may suggest a broader non pericyte role.

We performed a rescue of the *nkx3.1* mutant phenotype with *cxcl12* mRNA and previously showed that pericyte number is increased in both wildtype and mutant animals (significant increase in both cases). We have now added data to show that in cxcl12 mRNA rescue, hemorrhage is not significantly rescued (S12 Fig.).

Minor:

2-7. The authors need to give detailed information about how the brain pericytes are quantified, in terms of the region, software and other microscopy details.

We apologize for this omission. As per reviewer 1 query 1-6 we have updated these.

2-8. The rescue experiment in Fig. 5 should also include nkx3.1 +/-.

Due to the complexity breeding all of the genetic constructs (kdrl:mCherry; pdgfrb:Gal4; (or nkx3.1:Gal4); nkx3.1 mutant; UAS:cxcl12b), this experiment was done on homozygous progeny of homozygous parents (MZ)

animals. The point of the experiment is to show a rescue from the animals with the fewest pericytes (nkx3.1 MZ mutants). The control in this experiment is the nkx3.1 homozygote without the rescue construct. It is unclear what additional information a heterozygous animal would give.

Reviewer #3: The authors identify nkx3.1 positive precursor populations in the neural crest and head mesoderm which develop into brain pericytes.

They show that loss of nkx3.1 reduces pericyte numbers and causes brain hemorrhage. On the other hand, overexpression of nkx3.1 expression at 29-30 hpf increases pericyte numbers. They perform ScRNA seq on nkx3.1 positive cells and identify multiple cell populations, one which they claim is a pericyte precursor population but no experimental evidence is provided. The authors further explore the gene cxcl12b which is downregulated in nkx3.1 mutants. Inhibition of the signaling pathway reduces pericyte numbers and overexpression increases it. They also show that cxcl12b overexpression can promote pericyte development in embryos lacking pericytes just if the expression is driven by nxk3.1 and not if expression is driven by pdgfrb.

3-1. Statistical analysis of data. The smallest experimental unit is not the same between different analyses. Why is this the case, please explain. Are data normally distributed? Figure 1I, Fig 2C,- the smallest experimental unit is an experiment. How many individual embryos were analysed per each experiment? Figure 2F, 2I, Suppl. Figure 3C, Fig 4C, Fig 4F - the smallest experimental unit is an embryo.

We use different units when the underlying quantification measures different things. For instance, hemorrhage counts are graphed as experiments and not as individual embryos because the data is binary. Embryos either have a hemorrhage or not, and we count an entire clutch for hemorrhage to give a percentage hemorrhage in the clutch. Given that many embryos were used in this analysis, the proportion of embryos in a clutch with hemorrhage can be compared between different biological replicates. For this reason, we do 3 completely independent experiments (N=3; different days with different parents) and count the proportion of hemorrhage as a single data point for each independent experiment. The legend to figure 1l states that we used (N=3 experiments and 30 embryos (10 embryos/experiment)). For Figure 2C, we used (N=3, n=194 embryos). For S12 Fig, we used N=3, and a total of 141 uninjected and 167 injected animals.

All other statistics (other than fluorescent intensity) use pericyte counts from single embryos. This is because the counts within a single embryo are continuous (anywhere from 0-~30), and we can use the precision of discrete counts. We hope this answers these queries. These are standard ways of representing data in the field and we have published hemorrhage statistics and pericyte counts previously.

3-2. Figure 2E - the authors state that the Nxk3.1 deletion leads to "mispatterned brain vessels"? The authors should quantify the phenotype. From the provided image it is not obvious what is wrong.

Mispatterned or malformed are not precise terms and we should have avoided them. In the revision we add pericyte density (pericytes per vessel length), network length, and vessel diameter where appropriate to be more precise terms. We removed the word mispatterned.

3-3. Supplemental Figure 4 - The authors should also provide an image of control samples after 75 hpf showing blood vessels and Nxk3.1 expression.

In the manuscript revision, we have moved data from Supplemental figure 4 to the main figure 2. In the process, this panel was removed as it was meant to only show that vessels were still normal and did not match the data in Figure 2.

3-4. I am confused about the scRNA seq experiment and data interpretation. It is given that 500 embryos were used for scRNA seq - were all embryos analysed together? In different patches? The authors state that they had a little more than 3000 cells for analysis - this makes in average 6 cells sequenced per embryo.

Zebrafish embryos are small and there are very few nkx3.1 positive precursors. For instance, if you look at our pericyte counts there are 10-20/embryo at the 75 hpf timepoint. For scRNAseq, we are sampling at 30 hpf where there are likely fewer precursors as we show there is cell division of these cells between 48 and 75 hpf. 500 embryos are necessary in order to get ~3000 total nkx3.1 positive cells. These cells are from 500 siblings in two independent dissociations and sequencing. Dissociation from 30 hpf embryos is challenging as they are very delicate. Two quality control steps also removed cells as follows. Damaged cells and doublets are low quality cells and did not proceed from the FACS to the 10X pipeline. Then we also bioinformatically removed all the poor cells from the analysis (too few or too many UMIs, as described in the methods). What remains are high confidence cells, and we expect based on the number or pericytes that we see in older embryos, that we have sampled roughly 50% of the pericyte precursors per embryo.

The authors showed that both mesoderm and neural crest pericytes turn on nxk3.1 expression. What was the proportion of mesoderm and neural crest derived pericytes in isolated nxk3.1 positive cells? The cell cluster annotation should be explained in more detail. Could the authors explain basis on what evidence they conclude that some clusters contain pericyte precursors? - "Of the larger clusters, Prog-2, Fb-V and Fb-A express common fibroblast markers (col1a1a, col1a1b, pdgfrα, col5a1, mmp2; S5 Fig) suggesting that this group represents cells differentiating into fibroblast types, potentially including pericyte precursors." The markers Tbx18 and Cxcl12b used to define the cluster Fb-V as pericyte progenitor cluster could also suggest that this is a more general cluster for mural cells progenitors.

We cannot distinguish precursors originating from mesoderm and NCC in the clusters either bioinformatically (Fig 3), or by gene expression (See Fig 1 and S1 Fig, where there is one location of nkx3.1 expression in the head at multiple stages). This is why we suggest convergence of precursors from both embryonic origins to a singular pericyte phenotype/gene expression pattern in a single cluster (nkx3.1 precursors in Fig 6). All pericyte precursors appear identical in our analysis. We know that there are multiple origins of pericytes through lineage analysis (ours (S1 Fig) and others that are published, in fish and mouse). However, the lineage experiment is not easily quantitated, so we do not know the proportion that arises from each origin. Our work is consistent with the literature in that to our knowledge, no one has yet identified a marker that distinguishes a pericyte derived from NCCs and mesoderm once pericytes are differentiated.

We have no evidence in vivo using transgenics that nkx3.1 is expressed in SMC progenitors, which is why this is tentatively labelled a pericyte precursor cluster and not a mural cell cluster.

3-5 It is also very confusing that the cluster which according to authors contains pericyte precursors is named Fibroblast cluster (Fb-V). The reasoning why this cluster contains pericyte precursors is based on the expression of three additional genes and in my opinion, although supportive, is not confirmatory. Do mesoderm and

neural crest-derived pericytes develop via this common population found within Fb-V? How uniform is this Fb-V cluster? How do cells from individual embryos distribute in UMAP?

As we suggest above, we do not find evidence for separate clusters (or non-uniformity) of precursors that would suggest origins in mesoderm vs. NCC. In fact, no one has published any markers that distinguish mature pericytes by origin. This suggests that Nkx3.1 either unifies the pericyte transcriptional profile (since it is expressed at an early timepoint in pericytes of both origins and GOF induces more pericytes to be produced) or is expressed only in the unified population- we cannot distinguish. We opted to name these as Fibroblast subtypes as the large central cluster of nkx3.1 expressing cells in the scRNAseq that contains this cluster, also contains canonical fibroblasts, and it is clear that Fb-V is most related to fibroblasts, even thought they will go on to differentiate to pericytes. Through our lineage tracing experiments, we show that mesoderm and neural crest-derived pericytes are present in both mid- and hind-brain. Furthermore, our nkx3.1 GOF and LOF experiments has a consistent effect in both mid- and hind-brain indicating that Fb-V cluster marked by nkx3.1 contains pericytes derived from mesoderm and neural crest, which appear in the mid- and hind-brain.

3-6. How do the authors explain that after AMD3100 treatment brain pericyte numbers are reduced (Fig. 4F) but there is no change in vessel patterning (at least it is not mentioned by authors) (see Figure 2 E and F).

Note that the numbers of pericytes per brain are different between Fig 2 F and 4F (the same timepoint 75 hpf) - why? Also, why do pericyte numbers differ between 5C and 5F (filled circles).

The base number of pericytes in different experiments is an interesting observation. The AMD3100 experiment is done on control transgenic animals that are not derived from MZ nkx3.1 mutants. The numbers of pericytes in these animals is higher across all conditions. Embryos derived from MZ females have lower base pericyte numbers. Similarly, the base pericyte number is higher in the nkx3.1 GOF experiment, also done on wildtype transgenic. All other experiments were done in fish with a background MZ nkx3.1 deletion. For all other figures the comparator is MZ hets (the mother was homozygous mutant). We believe this explains the base numbers difference. As queried by the reviewer, we do not see any difference under any experimental condition including AMD3100 in vascular network length (S4 Fig).

3-6. Quantification of pericytes (fig 2F, 2I, 4F, Figure 5) - Please specify in the y-axis are shown pericyte numbers (correct?)

We have updated the graph axes.

3-7. Figure 6 - in my opinion the authors have not provided an evidence showing that mesoderm and neural crest-derived pericyte precursors merge into one fibroblast-like pericyte precursor.

We have two lines of experimental evidence that there is a single indistinguishable population of pericytes at the time of analysis. Firstly, the lineage analysis using both Sox10 and Tbx6 promoters resulted in labelling mid- and hindbrain pericytes, all of which identically express pdgfrb and nkx3.1 transgenes. Secondly, in the RNAseq analysis collected from Nkx3.1 pericyte precursors, there is only a single group of cells expressing markers later found in pericytes. To turn this argument around, you might ask whether we have any data to suggest there is more than one population, and the answer is no, we have no data to support the hypothesis that there is more than one population of pericyte precursors at the timepoint that nkx3.1 is expressed. Therefore, we have moved forward with the idea that at this timepoint, there is one population.

3-8. Discussion - "The only other gene that is sufficient to increase pericyte number is notch3, suggesting that Nkx3.1 and Notch3 may potentially act using similar downstream mechanisms" - Does nkx3.1 deletion affect notch3 expression?

This would be interesting, however in the RNAseq at 30 hpf did not detect any difference in notch3 expression in nkx3.1 mutants.

3-9. I could not find a description how pericytes were quantified and how was the cxcl12b expression (Fig 4C) expression quantified. These details should be added.

We apologize for this omission. As outlined in reply 1-6 above, we have updated the methods.

Minor points:

3-10 *Figure 1 D - for the clarity and guidance - please label hindbrain and midbrain regions.

Thanks for this suggestion, we have added brackets and MB/HB labels in Fig 1D.

3-11 *Supplemental Figure 1 - in which regions pericytes were quantified?

This figure gives examples of both the overlap with the indicated lineage tracers with the indicated transgenes in both hindbrain (first column) and midbrain (second column). Cells derived from lineages were found in both compartments. We have added detailed lineage analysis methods to the methods section as they were missing.
