# Supplementary material for: The development of brain pericytes requires expression of the transcription factor nkx3.1 in intermediate precursors
Source: PLoS Biol. 2024 Apr 29;22(4):e3002590. doi: 10.1371/journal.pbio.3002590 (PMC11081496; doi:10.1371/journal.pbio.3002590)
Supplement: Attachment — Submitted filename: Reply to reviews r2.docx [file pbio.3002590.s025.docx]

Reviewer #1: The authors have satisfactorily addressed all the concerns I originally raised.

However, I noticed that the Method section still lacks the descriptions of several important procedures, including the AMD3100 pharmacological treatment experiments (related to Fig. 4D-G) and the ablation experiments of nkx3.1-positive cells using the NTR-MTZ cell ablation method (related to Fig. 2M-Q).

The authors should include the detailed information on these missing experimental procedures in the Method section.

The catalog numbers and doses of AMD3100 and MTZ were already in Supplemental Table 2, and we have added a section to the drug treatments to explain the timing (24-48 hpf) and the medium that they were dissolved in (E3 fish water). We hope this is what the reviewer was looking for.

Reviewer #2: The authors made substantial improvements that have addressed many of the reviewer comments and have provided further data on the role of Nkx3.1-cxcl12b signalling in pericyte development.

*	Responses related to queries 2-2 and 2-5: The authors showed in the paper that Nkx3.1-cxcl12b signalling regulates brain pericyte development, however it is clear from their response that the phenotypes are broader and not exclusively specific to pericytes. The authors should clearly discuss this in the manuscript. Nkx3.1's broader role in fibroblasts and in different tissues need to be emphasized as well.

We have added the following sentence to the discussion. “Beyond the brain, previous work shows that *nkx3.1* is expressed in peri-vascular fibroblasts precursors of the trunk pericyte lineage (22). It is likely that *nkx3.1* is important for pericyte and fibroblast development in other areas of the embryo. Homozygous *nkx3.1* mutants are viable and fertile with reduced lifespan, suggesting that progeny of embryonic *nkx3.1*-expressing brain pericytes, trunk pericytes and/or fibroblasts likely contribute to progressive post-embryonic phenotypes.”

*	Related to query 2-8: To definitively interpret a rescue experiment, heterozygotes need to be present in the same experiment. This is needed to show that the mRNA rescue returns LOF phenotype to the equivalent of normal levels. Without this control, the definition may be limited to "an increase in the injected group compared to the nkx3.1-/- mutants" rather than a rescue.

We have gone through the text and changed ‘rescued’ for ‘increased’ and ‘not rescued’ to ‘does not change’.

*	FigS5: Fig legend defines animals as maternal-zygotic in the bold section, however in the further description says they are zygotic. Which is it? Also, C is missing stats on the graph.

Good catch- this was a typo. The supplemental data shows maternal zygotic mutants. We have included a statement in the text after this point to say “All work further therefore used maternal-zygotic *nkx3.1* mutants.”

*	Some minor points such as typos and stats need to be addressed by going through the manuscript and the figures thoroughly. Some examples are

o	Fig2C hemorrhage spelling, inconsistent font style and size, nkx3.1 spelling in the legend.

We have fixed both.

o	Last paragraph in the "Nkx3.1 function is required for brain pericyte development" was unclear due to potentially misplaced edits and incomplete sentences.

We edited these last few sentences and think that the sense is now clearer.

Reviewer #3: The authors have addressed some of the concerns raised in the previous review. However, the manuscript could still benefit from further clarification of experimental details and improved writing clarity. I apologize that some of my comments address points I missed in the first revised version, which I should have identified earlier.

I could not locate the Supplementary tables referenced in the manuscript, so I was unable to review these tables.

The supplementary tables, particularly S3 contain all raw primary data. We have pasted relevant tables into this document where appropriate below.

1. Abstract: Please consider rephrasing the first sentence in Abstract. The wording "regulating" implies influencing, not guaranteeing. Pericytes may regulate endothelial function in a way that promotes efficient blood flow under normal conditions, but they are not the only players. Blood flow regulation is a complex process affected by multiple factors. So, even if pericytes function optimally, other issues could still impair blood flow.

We think that the reviewer and the authors are all on the same page, pericytes are not the only player, but this doesn’t mean that they don’t regulate endothelial function, they are one of the players regulating function. We have rephrased to “Brain pericytes are one of the critical cell types”.

2. Page 5 "Pericytes in both hindbrain and midbrain express the nkx3.1 transgene (Fig. 1D)."

I find it difficult to identify nkx3.1-expressing cells in the midbrain region of your image. While you provided an enlarged image of the hindbrain region demonstrating nkx3.1 progeny, I would appreciate (and likely also other readers) a similarly enlarged image of the midbrain for clearer observation.

We have updated the image with separate subpanels for midbrain and hindbrain.

Additionally, your statement on page 5 regarding nkx3.1 expression by pericytes in the hindbrain /" Pericytes in both hindbrain and midbrain express the nkx3.1 transgene (Fig. 1D)"/ - The image in Fig. 1D depicts a 4 dpf stage, where nkx3.1 expression is no longer present. However, mCherry is present beyond nkx3.1 expression, and serves as an indicator of cells that previously expressed nkx3.1. Therefore, to accurately describe the observed data, the sentence should be rephrased accordingly.

This statement was found earlier on page 4, and we have made it more clear now: “mCherry perdures after native nkx3.1 mRNA is downregulated, allowing us to track cell lineage beyond the time that nkx3.1 mRNA is normally expressed”

3. S Fig1 - Please consider labelling "Midbrain" and "Hindbrain" and not "Mid Brain" and "Hind Brain"

This is done.

4. Fig. 2C-Please correct the misspelling - "hemmorage to "Hemorrhage".

Done.

5. In your response to a reviewer's comment, you mentioned a reduced lifespan for Nkx3.1-/- fish (1 year). Including this information directly in the main text (on page 6) would enhance clarity and provide valuable context. If possible, including quantitative data would further strengthen your claim.

This is updated.

6. Figure legend 2: "(E-F) Dorsal images of wildtype and nkx3.1 mutant embryo expressing Tg(pdgfrβ:GFP) and Tg(kdrl:mCherry) showing fewer brain pericytes (arrows) in mutants at 75 hpf." Please clarify which image (F or E) shows the mutant and which one wildtype?

We have rephrased this entire legend to make it clear which panels this was referring to. In the process we found an error in the statistics of Fig 2C and have corrected it. Thank you. We have also reworked Figure 4 and 5 legends to define panel numbers and controls in a similar way to reduce confusion.

Additionally, please specify whether you used wild-type or heterozygous embryos as controls in your experiment.

We agree this was not clear. Hets are controls in most panels, but wildtype are controls in the GOF panels. We have now made this explicit.

7. Figure 2FE, IJ and 4DE. I struggle to see differences between the pericyte numbers in the provided images. All images (2F vs. 2E and 2I vs. 2J and 4D vs. 4E) have an equal number of arrowheads. Despite all panels pointing to the same number of pericytes (four arrowheads), the authors claim panels 2E, 4E have fewer and panel 2J have more pericytes. This presentation format hinders clarity. I am unable to see these differences in the current figures. Please consider revising the data visualization for improved comprehension.

An excellent point. We have changed the number of arrows in the relevant panels in Figure 2 and Figure 4.

8. Supplementary Fig. 5 "There are significantly decreased brain pericyte numbers (C) and pericyte density (D) as compared to wildtype". The figure legend states comparison to wildtype data, but only heterozygote and knockout data are shown. Please include wildtype data or revise the legend to reflect the available data.

We have updated the legend to identify -/- mutants and +/- controls.

9. Scheme in Figure 3. - The figure legend lacks an explanation of what the schematic represents. Additionally, the meaning of the grey ovals is unclear. Please provide clarification in the legend.

The legend has been updated to state that they grey marks the eyes and this is a dorsal view of the entire zebrafish brain. We are sorry this image was not completely accessible to those outside the field. We hope this makes it clearer.

10. Response to reviewers, point 3.1.

While you mentioned this is the standard way of presenting data, I believe reaching an audience beyond zebrafish experts is valuable. Including all details about how the experiments were performed and data analysed would benefit them considerably. Additionally, providing these details in the Materials and Methods section aligns with transparency best practices and allows readers to fully understand the experimental procedures.

"Supplementary Figure 12 (G) Hemorrhage rates were unchanged in mutants at 48 hpf (N=3 with 141 uninjected at 167 injected mutants analyzed)" - Please report the individual embryo counts used in each experiment, rather than only providing the total number.

As the reviewer suggests, this information is in Supplemental Table 3, but now updated with embryo numbers for each replicate in addition to percentage that were graphed.

| **Column1** | **Embryo count** | **Hemorrhage count** | **%** |
| --- | --- | --- | --- |
| *nkx3.1-/-* | 45 | 14 | 31.1 |
| *nkx3.1-/-* | 62 | 15 | 24.2 |
| *nkx3.1-/-* | 34 | 14 | 41.2 |
| *nkx3.1-/-+cxcl12bmRNA* | 67 | 15 | 22.4 |
| *nkx3.1-/-+cxcl12bmRNA* | 56 | 14 | 25.0 |
| *nkx3.1-/-+cxcl12bmRNA* | 44 | 9 | 20.5 |

This raises another point: how were individual embryos selected for pericyte quantification? Did you specifically analyze those with hemorrhages, or exclude them? While the field might have standard practices like excluding hemorrhagic embryos, your Methods section lacks clarity on this aspect.

Embryos were sampled randomly from a clutch and included hemorrhaged and non-hemorrhaged embryos. The pericyte counts are reduced in both hemorrhaged vs. non-hemorrhaged -/- mutants. Hemorrhage occurs stochastically, with only a portion hemorrhaging at the time of sampling. This has been added to the methods.

11. Response to reviewers, point 3.4.

To ensure clarity, I recommend providing more detail in the Materials and Methods section regarding cell sorting for sequencing. Specifically, it is unclear whether cells were sorted in two separate rounds. Given that you generated two independent libraries, I assume this was the case. If so, please clarify the number of embryos used per preparation (250 per run?). Please show how cells from the two different batches (libraries) are distributed on the UMAP and give the percentage of cells from each batch in corresponding clusters.

We have updated the methods to specify that two biologically independent replicates were conducted, each with ~250 embryos (as many embryos as we could retrieve in a single day). We have added a new Figure S6 with UMAP overlay and proportions graph.

To analyze batch effects, the most notable differences between the samples are that the epidermal fibroblast (BasalFin cluster) is more highly represented in one sample, while Fb-B (more differentiated fibroblasts) are more represented in the other sample. We performed integrative analysis using Seurat to confirm whether these differences were due to batch effects. Our analysis suggested that these are not batch effects (i.e., they do not disappear with integration) are condition-specific clusters that arise from biological differences in sample preparation. We emphasize that even though the proportions are different in these two clusters, both clusters contain cells from both samples. Neither cluster is the focus of our manuscript. The potential sources of experimental variation include that the two samples were run 1.5 years apart by two different technical personnel, the embryos had different parents (same genotype for the mutant and transgenes used for sorting, but some variance expected from generation to generation). Further, the dissociation chemicals for the zebrafish used different batches/lots, meaning there may have been subtle variation in embryo digestion success, while FACs sorting parameters could also introduce variability.

I am also following up on my previous request to provide a more detailed explanation of the cluster annotation. Unfortunately, my previous comment about this has not been addressed. For example, "63% of the cells form a group of connected clusters that appear to be progenitors (Prog-1/2) and more differentiated cells" - to ensure clarity for readers unfamiliar with the specific field, I suggest providing additional details regarding the cluster annotations. Explaining the reasoning would greatly help readers understand your data and interpretation.

Thank you for this suggestion. We realized that the description of the clusters and assignment in the text was not linear and have worked on the text to make it far simpler to read. We have also updated Supp Table 1 with a new tab that lists the cluster assignment process including markers and references to scRNAseq resources that were used for cluster assignment. We believe this will make it much easier for the readers to understand the logic.

12. Mouse gene name should be written "Tbx18" (italics) and not "tbx18" (italics) (page 8).

This has been corrected.

13. Response to reviewers, point 3.9

Please provide a detailed description of the image acquisition for pericyte quantification. Include the numerical aperture of the objective.

This has been updated.

Please explain how you identified pericytes in the images. Please specify the precise region or area where pericytes were quantified. While citing reference 57 for the detailed method of image analysis, please provide a brief summary of how pericyte quantification (number and density) was performed.

We have expanded this method to explicitly say that pericytes must be transgene labelled and show a soma and processes. We also tell how the stack was dealt with to identify unique pericytes, and how the manual pericyte counts and automated vessel network length were integrated. Reference 57 is to a single cell paper. We do not reference a method, rather we reference the programs (ImageJ/Fiji; 64) and Vessel metrics (65)

Could you clarify what constitutes "erroneous segments"?

Automatic segmentation of highly angiogenic blood vessels will include angiogenic sprouts as part of the vessel skeleton because they express the transgene that we use for imaging. However, these non-lumenized sprouts are not yet connected, have no flow, and are not fully formed. We remove these from the network length to get an accurate pericyte density on lumenized vessels. In the VesselMetrics paper (https://doi.org/10.1016/j.mvr.2023.104610), erroneous segments are defined and dealt with as follows: “Fig. 4 shows erroneous burrs in red which are removed in post processing, including these burrs as additional vessel segments incorrectly inflates the number of segments within a region. We identified short terminal segments in the data by identifying segments thresholding with a short length and using the criteria that the short length segments were also associated only with only one branchpoint. A short terminal segment is a segment which only connects to one branch point and has a length *<*25 pixels; segments shorter than this tend to provide no useful information as they are so small that accurately characterizing vessel parameters is a challenge. These erroneous vessel segments are identified and removed.”

The pericyte density axis label needs clarification. What does the numerical value represent? Specify what unit this represents (e.g., pericytes per area unit, etc.).

This is defined on page 6 “(defined as the number of pericytes divided by the length of the vessel network)”. We have now added the same phrase to the legend of Figure 2 (the first time this metric appears in the paper) to make this simpler to understand.
